# Short-term risk stratification using parallel admission and reassessment features in PICU patients with infection

**DOI:** 10.3389/fped.2026.1834603

**Published:** 2026-06-04

**Authors:** Yuhang Wang, Cuie Chen, Guocheng Jiang, Chuanfu Xuan

**Affiliations:** Department of Pediatrics, Yiwu Maternity and Children Hospital, Yiwu, Zhejiang, China

**Keywords:** decision curve analysis, infection, machine learning, PICU, risk prediction

## Abstract

**Objective:**

To develop and validate an early risk prediction model for short-term major adverse events (MAE) in pediatric intensive care unit (PICU) admissions with infection using admission (M0) and early reassessment (M1) features.

**Methods:**

Using the PhysioNet Paediatric Intensive Care database, 684 infection-related PICU admissions were included in the final landmark-defined analytic cohort. The primary outcome was 72-hour MAE, defined as new vasoactive drug use, invasive mechanical ventilation, or death within 72 h after PICU admission. For the primary M0 + M1 analysis, prediction targeted events occurring after completion of the reassessment window. Features were extracted from M0 (0–6 h) and M1 (12–36 h). LASSO, random forest (RF), XGBoost, and a stacked ensemble were compared. Performance was assessed in an internal test cohort and a de-identified timestamp ordered validation cohort using AUC, PR-AUC, calibration metrics, Brier score, and decision curve analysis.

**Results:**

Among the candidate models, RF showed the most favorable overall numerical performance. In the internal test set, RF achieved an AUC of 0.724 and a PR-AUC of 0.741. In the de-identified timestamp–ordered validation cohort, the corresponding values were 0.718 and 0.766. Calibration was good in the internal test set but attenuated in the later cohort, suggesting the need for recalibration. SHAP analysis indicated complementary contributions of M0 and M1 features.

**Conclusions:**

A parallel M0 + M1 framework showed moderate discrimination and potential utility for reassessment-oriented early risk stratification in infection-related PICU admissions. Further external validation and recalibration are needed before broader application.

## Introduction

Infection-related admissions remain one of the most common and clinically heterogeneous reasons for pediatric intensive care unit (PICU) use. Among these children, clinical deterioration may occur early after admission despite initial stabilization, and major adverse events (MAE) such as vasoactive support, invasive mechanical ventilation, or death often reflect short-term progression rather than baseline severity alone. In this setting, the clinically relevant question is therefore not only which patients appear severely ill at presentation, but also which patients are likely to worsen over the ensuing hours after initial assessment and early management. Recent pediatric literature on deterioration prediction has similarly emphasized that risk assessment is stage-dependent and that models intended for early warning should be interpreted within the timing and context in which they are applied (1, 2).

In current PICU practice, conventional severity scores such as the Pediatric Risk of Mortality (PRISM) and Pediatric Index of Mortality (PIM) remain widely used for mortality prediction, benchmarking, and case-mix adjustment. However, their limitations should be considered more explicitly in relation to the present study question. These scores were primarily developed for broad severity assessment using admission-time or early fixed-window information, rather than for short-window prediction after initial management in a disease-focused cohort. In addition, subsequent evaluations have shown that their performance may vary across settings and selected subgroups, underscoring that these tools are not automatically transferable to all pediatric critical care contexts without renewed evaluation (3, 4). Relatedly, more recent ICU prediction work has shown that serially updated models can track risk evolution over time, supporting the broader rationale for time-structured rather than purely static representations of early critical illness (2).

Dynamic organ dysfunction assessment provides a related, but distinct, perspective. The pediatric Sequential Organ Failure Assessment (pSOFA) score has demonstrated prognostic relevance in critically ill children and has been associated with adverse outcomes or subsequent critical events. At the same time, recent studies also suggest that pSOFA alone does not function optimally as a stand-alone situational awareness or short-window early warning tool. It is therefore more appropriately understood as an organ dysfunction assessment framework than as a dedicated prediction model for impending 72-hour MAE after early PICU reassessment (5). More recently, the Phoenix criteria have been proposed to refine pediatric sepsis identification and classification in children with suspected infection (6). Although highly relevant to the contemporary pediatric sepsis field, Phoenix criteria address disease definition rather than the separate problem of individualized short-term risk prediction following early PICU assessment.

Against this background, the present study aimed to develop and validate an early risk prediction model for 72-hour MAE in PICU patients with infection using the Paediatric Intensive Care database available through the PhysioNet platform. Rather than relying solely on admission information, we evaluated whether preserving data from two clinically meaningful windows—initial assessment (M0) and early reassessment (M1)—could support short-term risk stratification within a prespecified training-testing framework. Model performance was assessed from the perspectives of discrimination, calibration, and potential clinical usefulness, with decision-curve analysis included as a brief complementary evaluation of threshold-dependent utility (7, 8).

Our working hypothesis was that representing M0 and M1 in parallel, rather than collapsing temporal information into simple deltas or ratios, would provide a more stable and clinically interpretable structure for early risk assessment. In this design, M0 reflects the patient's status at presentation, whereas M1 captures early physiologic evolution after initial management. This framework was intended to remain close to the routine clinical rhythm of initial assessment followed by reassessment, and to provide a practical basis for early risk communication and stratification in critically ill children with infection.

## Methods

### Study design and data source

This was a retrospective prediction model development and validation study based on the publicly available Paediatric Intensive Care (PIC) database hosted on the PhysioNet platform. The PIC database contains de-identified electronic health records from consecutive admissions to a tertiary pediatric intensive care unit, including demographic information, bedside vital signs, laboratory test results, diagnosis records, therapeutic interventions, and clinical outcomes. Access to the database was granted after completion of the required human-subjects protection training and approval under the PhysioNet data-use agreement. Because all records were de-identified before release, informed consent was not required, and the study was exempt from additional local ethics review in accordance with database governance requirements and the principles of the Declaration of Helsinki.

### Study population and definition of infection-related admissions

The analytic cohort was restricted to PICU admissions in children aged 0–18 years. For patients with more than one PICU stay, only the first eligible admission was retained. Infection-related admissions were identified using a prespecified rule-based algorithm that integrated diagnosis-text keywords with mapped ICD code prefixes. An admission was classified as infection-related if at least one diagnosis entry met either of the following criteria: the diagnosis text contained a predefined infection-related keyword, or the ICD code began with one of the mapped prefixes specified *a priori*. This operational definition was used to standardize cohort assembly in a large clinical dataset and was not intended to replace case-by-case etiologic adjudication. The full keyword list, ICD mapping rules, and operational notes are provided in Supplementary Text S1.

### Time windows and candidate predictor construction

To reflect the clinical sequence of initial assessment and early reassessment after PICU admission, two fixed time windows were defined *a priori* relative to the recorded admission time. The admission window (M0) was defined as 0–6 h after PICU admission, and the early reassessment window (M1) was defined as 12–36 h after admission. All timestamps were indexed to ICU admission time as time zero.

Event-level data were transformed into an admission-level analytic dataset with one row per admission. Window-specific variables were appended using the suffixes “_m0” and “_m1”. Candidate predictors were derived from three source domains: vital signs, laboratory tests, and blood gas measurements. Variable harmonization was performed using prespecified case-insensitive matching patterns to identify semantically similar raw labels across source tables. Detailed variable definitions, source domains, matching rules, window assignments, and units are provided in Supplementary Table S5.

Within each time window, repeated measurements were summarized according to prespecified clinically oriented aggregation rules rather than by simple averaging. Minimum values were used for variables in which lower values were considered to indicate greater physiologic compromise, such as peripheral oxygen saturation, pH, and oxygen tension. Maximum values were used for variables in which higher values were considered to reflect greater severity, such as heart rate, respiratory rate, inspired oxygen fraction, creatinine, blood urea nitrogen, partial pressure of carbon dioxide, and lactate. For variables intended to represent end-of-window biochemical status, including leukocyte count, inflammatory markers, albumin, transaminases, electrolytes, and bicarbonate, the last available measurement within the window was retained. If no measurement was available during a given window, the corresponding feature was treated as missing.

### Outcome definition

The primary outcome was post-landmark major adverse events (MAE) within 72 h of PICU admission, defined as the first occurrence after completion of the M1 window of any of the following: vasoactive drug initiation, invasive mechanical ventilation, or death within 72 h of PICU admission. For each admission, the earliest event time among the three MAE components was identified. A landmark framework was then applied so that the primary prediction target was MAE occurring after 36 h (the end of M1) and within 72 h of admission. Admissions in which any MAE occurred at or before the end of the M1 window were excluded from the primary M0 + M1 modeling analysis. In addition to the landmark-based primary endpoint, the pipeline also derived any 72-hour MAE irrespective of timing, as well as post-landmark invasive mechanical ventilation and post-landmark death variables for descriptive or ancillary use.

### Data cleaning and preprocessing

After construction of the window-specific predictors and definition of the landmark-eligible cohort, upstream cohort- and feature-level cleaning was performed before dataset partitioning to define the final analytic sample and candidate predictor set. This step included removal of candidate variables with more than 50% missingness, exclusion of admissions with more than 70% missingness across the retained candidate predictors, elimination of constant or near-zero variance predictors, and collinearity filtering based on pairwise correlations. When the absolute correlation coefficient between two continuous variables was at least 0.90, one variable was removed, preferentially retaining the more clinically interpretable variable or the variable with less missingness. These upstream cleaning steps were based only on variable availability and the distributional structure of candidate predictors; outcome labels were not used, and no model parameters were estimated at this stage.

After dataset partitioning, all model-level preprocessing parameters were estimated exclusively within the training data and then applied unchanged to the internal test set and the de-identified timestamp–ordered validation set. Continuous predictors were processed using an age-band-aware preprocessing pipeline. Specifically, missing numeric values were imputed using age-band-specific medians derived from the training set; continuous variables were winsorized within age bands at the 1st and 99th percentiles using training-set quantiles; and standardization was performed using the training-set mean and standard deviation. Age band was one-hot encoded according to the categories observed in the training data and retained as a fixed covariate in all models. Age bands were defined as 0–1, 1–3, 3–6, 6–12, and 12–18 years. A detailed preprocessing and exclusion log is provided in Supplementary Table S7.

### Model development

The primary modeling strategy preserved M0 and M1 predictors as separate variables in order to retain the clinical meaning of each assessment stage. Delta variables and ratio-based transformations were not included in the primary specification because they can amplify measurement noise and obscure the temporal interpretation of predictor effects.

Four candidate models were developed and compared: logistic regression with L1 regularization (LASSO), random forest, extreme gradient boosting (XGBoost), and a stacked ensemble. The stacked model used logistic regression as the meta-learner and combined predictions from the LASSO, random forest, and XGBoost base models. Hyperparameter tuning was performed within the training data using prespecified search grids and five-fold stratified cross-validation, with mean precision–recall area under the curve as the tuning objective. Model-specific search spaces and final selected parameter settings are provided in Supplementary Table S6. Class imbalance was handled using model-appropriate weighting strategies, including class_weight=“balanced” for LASSO and random forest and a training-set-derived scale_pos_weight term for XGBoost. All models generated probabilistic predictions for downstream performance evaluation.

### Model evaluation and validation

The landmark-eligible derivation dataset was divided into training and internal test sets using stratified random sampling in a 70:30 ratio. All preprocessing, tuning, and model fitting procedures were confined to the training set, whereas the held-out test set was used only for model comparison.

The landmark-eligible analytic cohort was first sorted according to the de-identified ICU admission timestamp. The latest quarter of admissions in this ordering was reserved as a timestamp–ordered validation cohort, and the remaining admissions constituted the derivation cohort. Within the derivation cohort only, a stratified random 70:30 split was then performed to create the internal training and internal test sets. All preprocessing, hyperparameter tuning, and model fitting procedures for internal model comparison were confined to the internal training set, whereas the held-out internal test set was used only for model comparison.

Because dates in the PIC database are de-identified by patient-specific shifting, the timestamp–ordered validation split was used to assess model transportability across the preserved within-database temporal ordering rather than to represent actual calendar periods.

Model discrimination was assessed using the area under the receiver operating characteristic curve (AUC) and the area under the precision–recall curve (PR-AUC). Calibration was assessed using calibration plots, the Brier score, and calibration intercept and slope. Clinical utility was examined using decision-curve analysis across threshold probabilities of 0.10–0.50. Threshold-dependent operating characteristics were additionally summarized at selected cutoffs of 0.10, 0.20, 0.30, 0.35, 0.40, and 0.50. Uncertainty in key performance metrics was quantified by bootstrap resampling (500 iterations) to derive 95% confidence intervals.

### Model interpretation

For the best-performing model, predictor contributions were examined using Shapley additive explanations (SHAP). Predictors were ranked according to their mean absolute SHAP values, and the relative contributions of M0 and M1 features were compared to characterize the balance between baseline severity signals and early reassessment information. SHAP findings were interpreted as model-based explanations of prediction behavior rather than as evidence of causal effects.

### Statistical software

All data management, preprocessing, model development, performance evaluation, and visualization procedures were conducted in Python 3.13.5 using pandas 2.2.3, numpy 2.3.5, scikit-learn 1.8.0, xgboost 3.1.3, shap 0.50.0, and matplotlib 3.10.8. Random operations used a fixed seed of 123. The complete consolidated analysis workflow is provided as Supplementary Code S1.

## Results

A total of 684 admissions were included in the final landmark-defined analytic cohort for the primary M0 + M1 analysis. Of these, 301 (44.0%) did not experience a landmark-defined major adverse event (MAE) during the prespecified 36–72 h prediction interval after PICU admission, whereas 383 (56.0%) did. As shown in Table 1, compared with the non-MAE group, admissions in the MAE group were characterized by lower minimum SpO₂ values and higher maximum heart rate and maximum respiratory rate across both time windows. For laboratory variables, AST, total bilirubin, creatinine, and BUN were generally higher in the MAE group, with between-group differences generally more pronounced in the M1 window. In blood gas measurements, the MAE group showed higher pCO₂ and lower pO₂, and these differences were more evident in M0. By contrast, inflammatory markers showed less consistent separation than respiratory, biochemical, and blood gas variables. WBC, CRP, and lymphocyte percentage did not demonstrate stable between-group differences across the two windows, and PCT in M0 did not show a consistent between-group difference. Age stratification further showed that infants aged 0–1 year accounted for a greater proportion of the MAE group than of the non-MAE group (70.8% vs. 57.8%, *P* = 0.004).

**Table 1:** Baseline characteristics and window-specific candidate predictors of the landmark-analysis cohort according to major adverse events occurring between 36 and 72 h after PICU admission.

Variable	Total (*n* = 684)	MAE=0 (*n* = 301)	MAE=1 (*n* = 383)	*P* value
Demographic characteristics				
Age band, *n* (%)				0.004
0–1 year	445 (65.06)	174 (57.81)	271 (70.76)	
1–5 years	147 (21.49)	81 (26.91)	66 (17.23)	
5–12 years	72 (10.53)	37 (12.29)	35 (9.14)	
12–18 years	20 (2.92)	9 (2.99)	11 (2.87)	
Admission vital signs (M0)				
SpO₂ min M0 (%)	98.37 ± 1.84	98.77 ± 1.38	98.05 ± 2.08	<0.001
HR max M0 (bpm)	136.72 ± 16.70	135.00 ± 17.90	138.07 ± 15.58	0.019
RR max M0 (breaths/min)	39.49 ± 11.74	36.15 ± 9.47	42.12 ± 12.65	<0.001
Reassessment vital signs (M1)				
SpO₂ min M1 (%)	98.78 ± 1.11	98.92 ± 0.74	98.66 ± 1.32	0.003
HR max M1 (bpm)	137.73 ± 19.36	135.23 ± 19.84	139.70 ± 18.76	0.003
RR max M1 (breaths/min)	42.90 ± 18.60	38.70 ± 15.20	46.20 ± 20.60	<0.001
Inflammatory markers				
WBC M0 (×10⁹/L)	10.48 ± 7.68	9.86 ± 7.27	10.96 ± 7.97	0.062
WBC M1 (×10⁹/L)	10.10 ± 8.60	9.67 ± 7.16	10.44 ± 9.58	0.230
CRP M0 (mg/L)	17.63 ± 29.34	15.45 ± 26.93	19.34 ± 31.03	0.085
CRP M1 (mg/L)	26.32 ± 33.18	24.07 ± 31.27	28.08 ± 34.54	0.116
PCT M0 (ng/mL)	0.25 ± 0.14	0.27 ± 0.15	0.23 ± 0.13	<0.001
PCT M1 (ng/mL)	0.87 ± 3.57	0.93 ± 3.71	0.83 ± 3.46	0.716
Renal and liver function				
Albumin M0 (g/L)	31.6 ± 5.4	30.9 ± 5.2	32.1 ± 5.5	0.003
Albumin M1 (g/L)	32.1 ± 5.8	31.4 ± 5.6	32.6 ± 5.9	0.011
ALT M0 (U/L)	29.91 ± 19.84	29.99 ± 19.37	29.85 ± 20.23	0.926
ALT M1 (U/L)	35.8 ± 29.6	32.9 ± 26.4	38.1 ± 31.6	0.028
AST M0 (U/L)	46.78 ± 11.35	45.33 ± 11.51	47.92 ± 11.10	0.003
AST M1 (U/L)	61.5 ± 46.2	55.9 ± 40.8	65.9 ± 49.3	0.004
Total bilirubin M0 (µmol/L)	53.72 ± 52.85	45.91 ± 45.31	59.86 ± 57.42	<0.001
Total bilirubin M1 (µmol/L)	44.10 ± 51.85	33.02 ± 41.33	52.80 ± 57.39	<0.001
Creatinine M0 (µmol/L)	36.99 ± 15.69	34.84 ± 9.84	38.69 ± 18.91	0.001
Creatinine M1 (µmol/L)	53.19 ± 22.99	48.29 ± 19.27	57.05 ± 24.89	<0.001
BUN M0 (mmol/L)	3.37 ± 1.42	3.24 ± 1.06	3.46 ± 1.64	0.041
BUN M1 (mmol/L)	4.11 ± 2.62	3.60 ± 1.84	4.52 ± 3.05	<0.001
Blood gas and metabolic markers				
pH M0	7.37 ± 0.10	7.38 ± 0.09	7.36 ± 0.10	<0.001
pH M1	7.34 ± 0.08	7.35 ± 0.07	7.33 ± 0.09	0.002
pCO₂ M0 (mmHg)	41.68 ± 13.11	39.60 ± 11.70	43.32 ± 13.92	<0.001
pCO₂ M1 (mmHg)	40.34 ± 10.54	38.75 ± 8.94	41.60 ± 11.50	<0.001
pO₂ M0 (mmHg)	114.78 ± 55.56	128.56 ± 57.40	103.95 ± 51.63	<0.001
pO₂ M1 (mmHg)	116.22 ± 49.09	120.29 ± 49.25	113.01 ± 48.78	0.054
Lactate M0 (mmol/L)	2.61 ± 2.73	2.27 ± 2.70	2.88 ± 2.72	0.004
Lactate M1 (mmol/L)	4.7 ± 2.3	3.9 ± 1.9	5.3 ± 2.5	<0.001

MAE, major adverse event; HR, heart rate; RR, respiratory rate; WBC, white blood cell count; CRP, C-reactive protein; PCT, procalcitonin; ALT, alanine aminotransferase; AST, aspartate aminotransferase; BUN, blood urea nitrogen.

The final analytic cohort was then divided according to the de-identified ICU admission timestamp ordering into a derivation cohort of 513 admissions, including 287 landmark-defined MAE events, and a de-identified timestamp–ordered validation cohort of 171 admissions, including 96 events. Within the development cohort, 359 admissions with 201 events were used for model training and 154 admissions with 86 events were reserved for internal testing.

**Figure 1:**
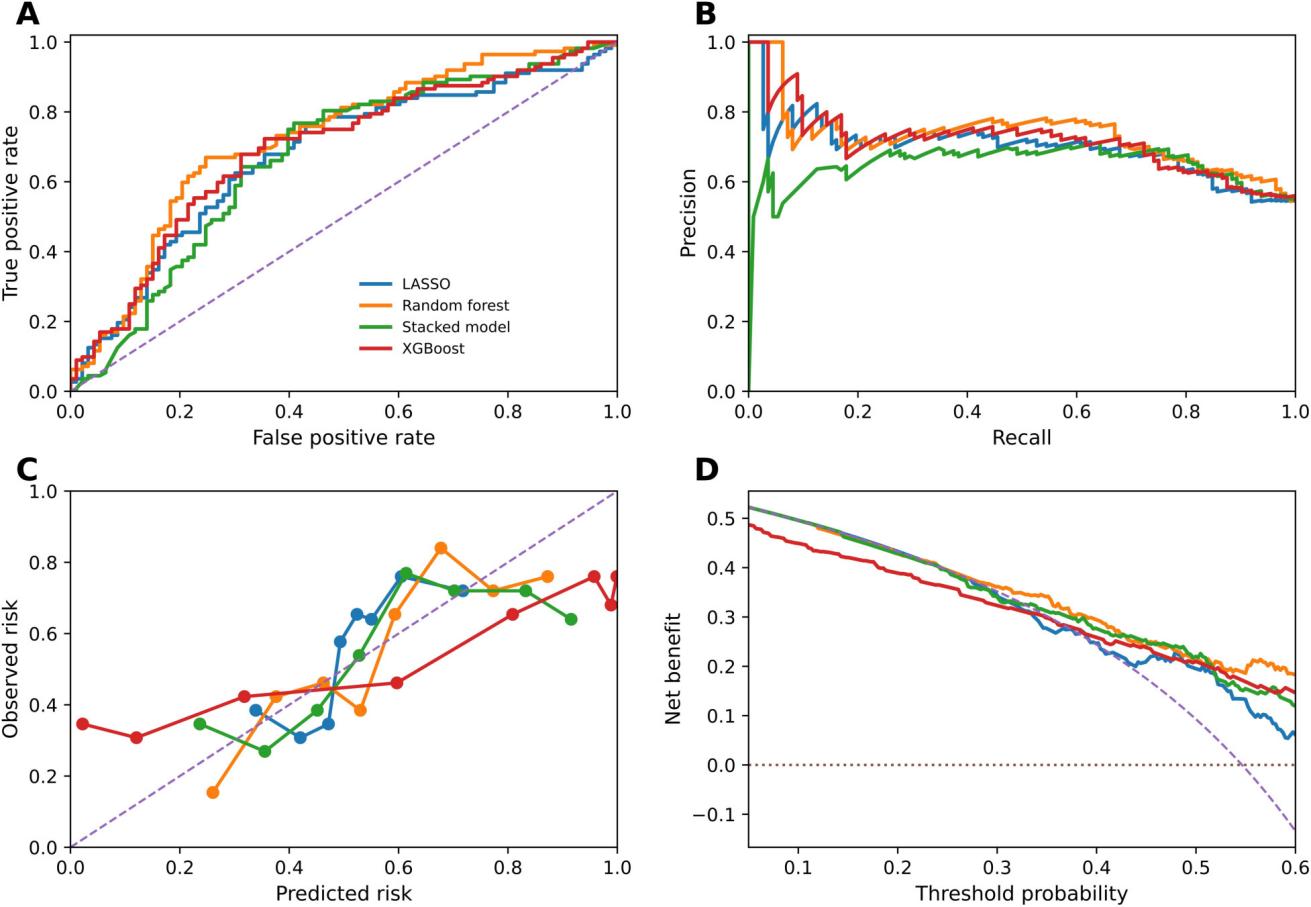
Internal test performance of the four candidate models for landmark-defined major adverse events occurring between 36 and 72 h after PICU admission using the combined M0 + M1 feature set. **(A)** Receiver operating characteristic curves of LASSO, random forest, stacked ensemble, and XGBoost. **(B)** Precision–recall curves of the four candidate models. **(C)** Calibration plots comparing predicted and observed risk across the four models. **(D)** Decision curve analysis of the four candidate models. Colored solid lines indicate model-based net benefit, the dashed line indicates the treat-all strategy, and the dotted horizontal line indicates the treat-none strategy. Interpretation focuses on threshold probabilities from 0.10 to 0.50.

In the internal test set, all candidate models showed acceptable-to-moderate discriminatory performance, with AUCs ranging from 0.675 to 0.724 and PR-AUCs ranging from 0.690 to 0.741 (Figures 1A, B). Among the evaluated models, the random forest model showed the most favorable overall numerical performance in the present dataset, with an AUC of 0.724 (95% CI 0.645–0.803) and a PR-AUC of 0.741 (95% CI 0.661–0.812). The corresponding AUCs were 0.704 (95% CI 0.623–0.785) for the stacked ensemble, 0.693 (95% CI 0.611–0.775) for XGBoost, and 0.675 (95% CI 0.591–0.759) for LASSO, while the corresponding PR-AUCs were 0.690 (95% CI 0.612–0.761), 0.702 (95% CI 0.624–0.774), and 0.697 (95% CI 0.619–0.769), respectively. Calibration analysis showed that the random forest model had the lowest Brier score (0.214), with a calibration intercept of −0.02 and a calibration slope of 0.95. The stacked ensemble showed a broadly comparable calibration profile, whereas LASSO and XGBoost displayed more visible deviation from the ideal line across parts of the risk spectrum (Figure 1C). In decision curve analysis, the random forest and stacked ensemble models yielded numerically greater and more stable net benefit across threshold probabilities of approximately 0.10–0.30, whereas the other models showed greater fluctuation (Figure 1D).

**Figure 2:**
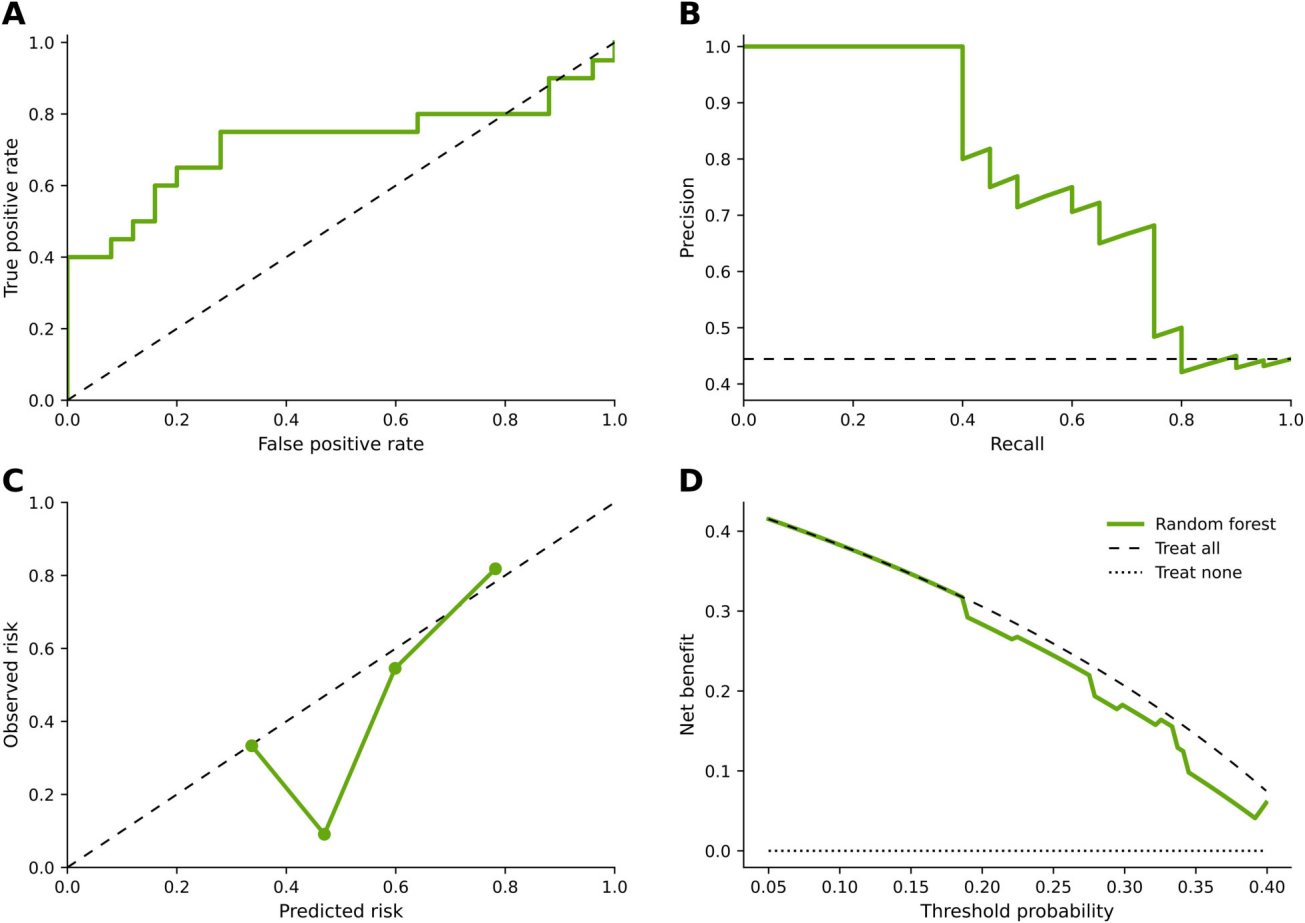
Temporal validation performance of the random forest model for landmark-defined major adverse events occurring between 36 and 72 h after PICU admission. **(A)** Receiver operating characteristic curve of the random forest model in the timestamp ordered validation cohort. **(B)** Precision–recall curve of the random forest model in the timestamp ordered validation cohort. **(C)** Calibration plot showing predicted vs. observed risk in the timestamp ordered validation cohort. **(D)** Decision curve analysis of the random forest model. The solid green line indicates model-based net benefit, the dashed line indicates the treat-all strategy, and the dotted horizontal line indicates the treat-none strategy. Interpretation focuses on threshold probabilities from 0.10 to 0.50.

The selected random forest model was then evaluated in the de-identified timestamp–ordered validation cohort. In this cohort, the model maintained similar discriminatory performance, with an AUC of 0.718 (95% CI 0.642–0.794) and a PR-AUC of 0.766 (95% CI 0.691–0.838) (Figures 2A, B). The corresponding Brier score was 0.221, with a calibration intercept of −0.09 and a calibration slope of 0.82. As shown in Figure 2C, calibration was less uniform in the de-identified timestamp–ordered validation cohort, with more apparent deviation from the ideal line in the intermediate-risk range, suggesting modest calibration drift. Decision curve analysis further showed that, across the evaluated threshold range, the model consistently provided positive net benefit relative to the treat-none strategy, whereas additional benefit over the treat-all strategy was mainly confined to lower threshold probabilities (Figure 2D).

**Figure 3:**
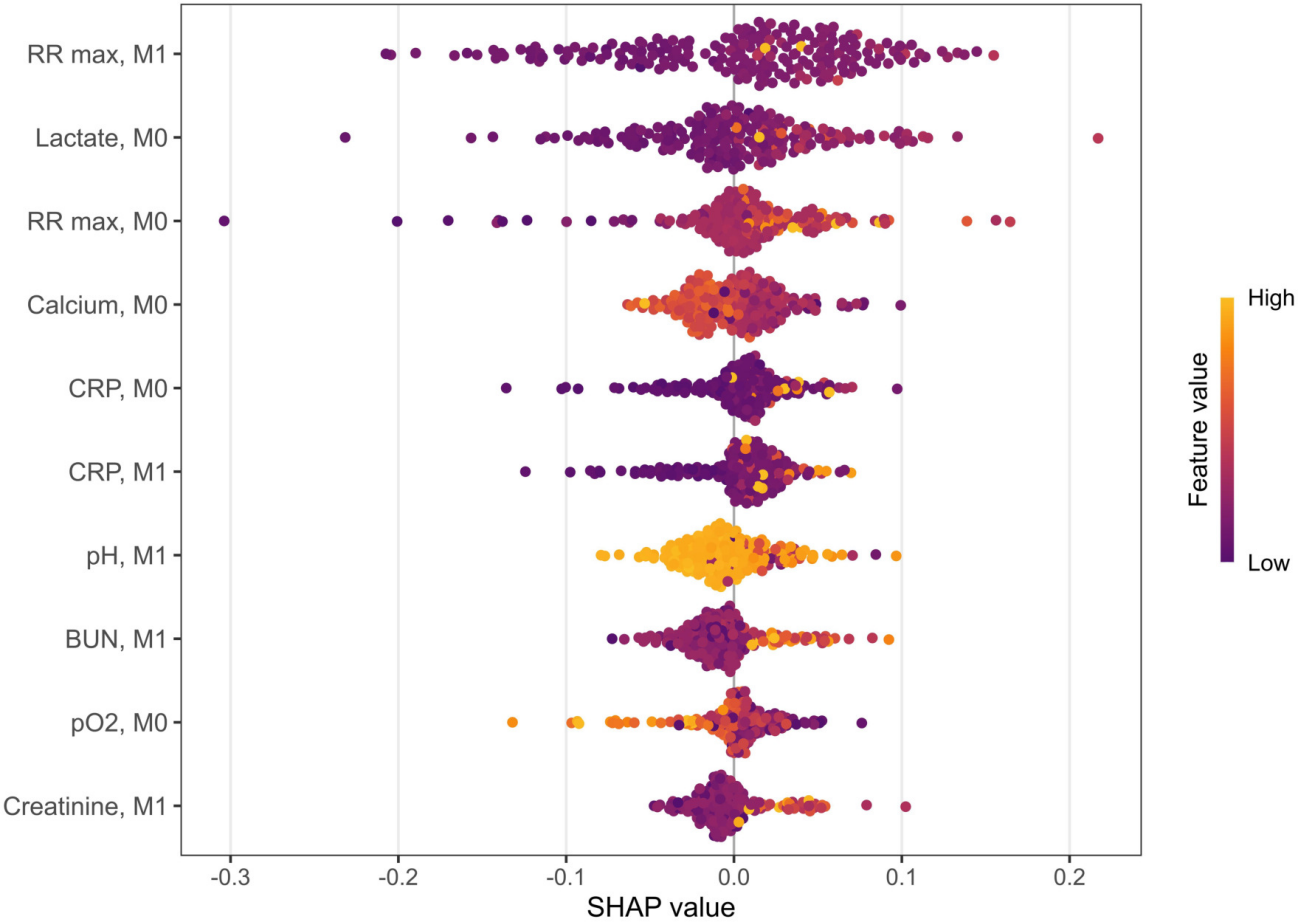
SHAP beeswarm summary plot of the random forest model for landmark-defined major adverse events occurring between 36 and 72 h after PICU admission using the combined M0 + M1 feature set. Each point represents one child. The horizontal position indicates the SHAP value for the corresponding feature, and color represents the original feature value. Positive SHAP values indicate increased predicted risk of landmark-defined post-M1 major adverse events, whereas negative SHAP values indicate reduced predicted risk. M0 denotes admission-window features and M1 denotes early reassessment-window features.

The SHAP beeswarm plot for the random forest model is shown in Figure 3. The highest-ranking features in the combined M0 + M1 feature set were RR max (M1), Lactate (M0), RR max (M0), Calcium (M0), CRP (M0), CRP (M1), pH (M1), BUN (M1), pO₂ (M0), and Creatinine (M1). These features spanned both the admission and early reassessment windows and represented several physiological domains, including respiratory status, metabolism, inflammation, blood gas parameters, and renal function. In the beeswarm distribution, higher values of RR max, lactate, and CRP, together with lower values of pH and pO₂, were generally associated with higher predicted landmark-defined MAE risk. Respiratory rate appeared among the highest-ranking features in both M0 and M1, whereas lactate, calcium, and pO₂ were more prominent in the admission window, and pH, BUN, and creatinine were more prominent in the reassessment window. These findings are presented as model-based associations that help interpret the fitted random forest model and should not be taken as evidence of causal effects.

## Discussion

In this study, we evaluated a parallel-window modeling strategy that preserved both admission information (M0) and early reassessment information (M1) in PICU admissions with infection. Several findings merit emphasis. The combined M0 + M1 framework achieved moderate discrimination in both the internal test set and the de-identified timestamp–ordered validation cohort, suggesting that short-term deterioration risk in this setting can be meaningfully characterized using routinely available early data collected across two clinically relevant windows. Among the candidate models, the random forest model showed the most favorable overall numerical profile, with relatively balanced discrimination, calibration, and decision-curve performance. In addition, the most influential predictors were distributed across multiple physiologic domains and across both windows, supporting the view that early major adverse events in infection-related critical illness are more likely to be captured by dynamic multisystem information than by any single variable considered in isolation.

These findings are clinically plausible. In the present cohort, admissions that developed landmark-defined MAE showed broader abnormalities in oxygenation, respiratory burden, renal function, bilirubin metabolism, and selected blood gas indices, whereas inflammatory markers showed less consistent univariable separation. The model-based feature pattern was broadly concordant with this clinical picture. Rather than depending on one dominant signal, the fitted model appeared to integrate respiratory, metabolic, inflammatory, acid-base, oxygenation, and renal information. Such a pattern is consistent with the multidimensional nature of early deterioration in pediatric critical infection, in which instability often emerges from the interaction of several organ systems rather than from a single pathway.

Compared with earlier work based on composite severity scores, the present study adopted a narrower and more explicitly time-structured approach. PRISM III and PIM were developed primarily for mortality risk assessment in broad PICU populations and have subsequently been used widely for benchmarking and risk adjustment rather than for short-window deterioration assessment in a disease-focused cohort (9–11) Although these tools remain important reference standards, later studies have also shown that their calibration may vary across settings and may require local reevaluation or recalibration before clinical interpretation (11). Recent pediatric data further suggest that repeated organ dysfunction assessment may outperform single early summary scores in outcome prediction, and pSOFA reassessment has been reported to show stronger prognostic performance than PRISM III and PIM2 in some cohorts (12). Taken together with earlier ICU literature on serial assessment (13). these observations support the rationale for preserving both the initial evaluation and the early reassessment as separate representations of early critical illness, rather than collapsing them into a single static summary.

The rationale for using M0 and M1 in parallel is also methodologically relevant. Dynamic information is intuitively attractive in critical care, but the way it is encoded matters. Delta-based or ratio-based constructs are concise, yet they may be sensitive to asynchronous sampling, biologic noise, and denominator instability. By contrast, the present parallel-window approach retains the clinical meaning of each time point and allows the model to weigh admission and reassessment variables separately. This structure also aligns more closely with clinical reasoning in pediatric deterioration assessment, where risk interpretation is embedded in context rather than divorced from workflow (14). In pediatric sepsis care, repeated assessment and timely response to evolving physiology are likewise emphasized in international guidance, making a reassessment-oriented framework clinically more natural than a purely admission-only representation (15).

The SHAP results were consistent with this interpretation, although they should be understood strictly as model-based associations rather than as evidence of mechanism. The most influential features included maximum respiratory rate in both windows, lactate at M0, calcium at M0, CRP in both windows, pH at M1, BUN at M1, pO₂ at M0, and creatinine at M1. This pattern suggests that the model was drawing jointly on presentation severity and early response or nonresponse over the next observation period. In particular, the prominence of lactate, CRP, and renal markers is broadly compatible with prior pediatric sepsis literature showing that lactate-based and inflammatory markers may carry prognostic information in critically ill children, although no single biomarker is sufficient on its own (16). The current findings therefore support a complementary rather than hierarchical interpretation of M0 and M1.

From a clinical utility perspective, the decision-curve findings also deserve a restrained interpretation. In the present study, the selected model retained positive net benefit relative to a treat-none strategy across the evaluated threshold range, whereas additional benefit over treat-all was concentrated mainly at lower thresholds. This pattern does not support a single universal cutoff. Instead, it suggests that the model may be more useful as a reassessment-oriented prioritization aid within a defined operating range. This interpretation is consistent with the logic of decision-curve analysis itself, which is explicitly threshold dependent (17), and with the broader principle that calibration materially affects decision-analytic value even when discrimination appears acceptable (18). In practice, any escalation threshold would still need to reflect local staffing, monitoring capacity, and tolerance for false-positive alerts.

This point is especially relevant in contemporary sepsis screening environments. Pediatric sepsis screening practices vary substantially across hospitals (19), and implementation studies in PICU settings have highlighted the importance of workflow integration, clinician trust, and the burden imposed by poorly aligned alerts (20). In that context, the present model is probably best understood not as an automated decision-maker, but as a structured aid to bedside prioritization, reassessment, and risk communication after the earliest stabilization period.

Several strengths should be acknowledged. First, the cohort was restricted to infection-related PICU admissions, which narrowed the clinical context relative to general PICU case-mix studies and allowed the analysis to address a more focused prognostic question. Second, predictor construction was explicitly organized around two clinically interpretable windows, which preserved temporal meaning. Third, model evaluation was not limited to discrimination, but also incorporated calibration and decision-curve analysis, yielding a more complete picture of potential performance. Finally, evaluation in the de-identified timestamp–ordered validation cohort suggested that the overall performance pattern was not confined entirely to the derivation cohort, although absolute risk estimates were less stable in the validation cohort.

The study also has important limitations. It was based on a single-center public database, and true geographic or institutional external validation was not available. Infection classification relied on a prespecified rule-based operational definition integrating diagnosis text and mapped ICD prefixes, so some residual misclassification cannot be excluded. In addition, predictor construction depended on the availability and timing of routine clinical measurements within the M0 and M1 windows. As a result, inclusion in the analytic sample was partly conditioned on testing patterns, and more intensively monitored admissions may have been overrepresented. Although imputation and sensitivity checks can improve robustness, they cannot fully remove bias when measurement probability is related to severity.

A further limitation arises from the landmark-defined design itself. The primary analysis excluded admissions in which MAE occurred before completion of the M1 window. This improved temporal ordering between reassessment features and subsequent outcomes and reduced the risk of post-event information leakage, but it also means that the model was not intended to identify the very earliest deterioration events. Its most appropriate role is therefore reassessment-oriented risk stratification among admissions that remain event-free through the initial observation period, rather than universal admission-time prediction. The moderate discrimination observed in both internal testing and timestamp–ordered validation also suggests that the predictive ceiling of a model based on routinely collected early variables may be inherently limited. Moreover, calibration was less stable in the de-identified timestamp–ordered validation cohort, indicating that performance may drift over time and that recalibration should be considered before application in other settings or future datasets (21).

Future work should therefore focus on transportability and implementation rather than on incremental algorithmic complexity alone. Multicenter validation across geographically and operationally distinct PICUs will be necessary to determine how stable this two-window framework remains under different monitoring practices and pathogen spectra. Prospective implementation studies will also be needed to determine whether a reassessment-oriented model can improve escalation timeliness or resource prioritization without creating excessive alert burden. Finally, incorporation of richer physiologic trajectories, treatment-response variables, and recalibration strategies may improve performance, but such efforts should preserve clinical interpretability and explicitly account for calibration drift over time.

## Conclusions

In PICU patients with infection, a parallel-window model that simultaneously represented admission (M0) and early reassessment (M1) features showed moderate discriminatory ability and retained clinically relevant net benefit in both internal testing and timestamp–ordered validation. By preserving separate representations of baseline status and early physiologic evolution, this framework provides an interpretable basis for reassessment-oriented early risk stratification. However, calibration was less stable in the timestamp–ordered validation cohort, indicating that recalibration may be necessary before broader application. Overall, these findings support the parallel-window approach as a practical adjunct for bedside reassessment and early risk communication in pediatric critical infection.

## Data Availability

Publicly available datasets were analyzed in this study. This data can be found here: http://pic.nbscn.org/.
