# Supplementary material for: Short-term risk stratification using parallel admission and reassessment features in PICU patients with infection
Source: Front Pediatr. 2026 Jun 4;14:1834603. doi: 10.3389/fped.2026.1834603 (PMC13295176; doi:10.3389/fped.2026.1834603)
Supplement: Supplementary file 1 [file Table1.docx]

Supplementary Text S1. Infection keyword list and ICD mapping rules

Purpose

To improve the transparency, reproducibility, and consistency of cohort assembly, infection-related admissions were identified using a prespecified rule-based screening algorithm that integrated diagnosis text keywords with ICD code prefixes. This operational definition was used for standardized case ascertainment in the study database and was not intended to replace individual clinical adjudication.

Selection rule

An admission was classified as infection-related when at least one diagnosis record met either of the following prespecified criteria:

(1) the diagnosis text contained at least one infection-related keyword; or

(2) the ICD code began with one of the mapped ICD prefixes listed below.

This dual approach was adopted because reliance on diagnosis text alone may miss structured coding information, whereas use of ICD codes alone may fail to capture clinically relevant diagnoses recorded only in free text. By combining both sources, the screening procedure aimed to improve case capture while maintaining explicit and reproducible rules.

Diagnosis text keyword list

The following infection-related keywords were used for free-text screening of diagnosis records:

- pneumonia

- bronchiolitis

- sepsis

- septic

- meningitis

- encephalitis

- cellulitis

- pyelonephritis

- urinary tract infection

- uti

- bacteremia

- fungemia

- candidemia

- influenza

- rsv

- adenovirus

- mycoplasma

- pertussis

- covid

- infection

- infectious

- abscess

- empyema

- peritonitis

- gastroenteritis

- enterocolitis

- osteomyelitis

- septic arthritis

- viral

- bacterial

- fungal

- parasitic

- kawasaki

Keyword matching rule

Keyword matching was performed in a case-insensitive manner. A diagnosis record was considered text-positive if any prespecified keyword appeared in the diagnosis text. The keyword list was designed to cover common infectious syndromes, pathogen-related terms, and generic infection descriptors encountered in routine pediatric documentation.

ICD mapping prefixes

The following ICD code prefixes were used to identify admissions with structured diagnosis codes compatible with infectious disease or closely related infection-specific conditions:

- A

- B

- G00

- G03

- J

- N10

- N12

- L03

- K35

- K65

- M00

- M86

- P36

- R65

Interpretation of mapped ICD prefixes

The mapped prefixes were selected to capture major infectious disease categories and infection-specific diagnoses commonly encountered in pediatric inpatient care. In particular, prefixes A and B cover infectious and parasitic diseases. Prefixes G00 and G03 capture meningitis-related diagnoses. Prefix J was used to capture respiratory diagnoses and was interpreted in conjunction with the study’s keyword-based screening framework rather than as an isolated stand-alone confirmation of respiratory infection severity. Prefixes N10 and N12 represent kidney infection-related diagnoses, L03 captures cellulitis, K35 and K65 represent appendicitis/peritonitis-related abdominal infection contexts, M00 and M86 capture septic arthritis and osteomyelitis, P36 captures neonatal sepsis, and R65 captures systemic inflammatory response or sepsis-related severity coding.

Operational notes

This algorithm was designed as a prespecified screening tool for identifying infection-related admissions in a large clinical dataset. It was intended to standardize cohort assembly and reduce subjective variability in manual selection. Because administrative codes and free-text diagnosis entries may each contain omissions or inconsistencies, the rule combined both sources to improve ascertainment. The resulting infection cohort should therefore be interpreted as being based on an operational database definition.

Limitations

This rule-based strategy may still be subject to misclassification. Some broad terms in diagnosis text may capture heterogeneous infectious presentations, and some ICD prefixes may include conditions that require clinical context for exact interpretation. Accordingly, this algorithm was used to support standardized study inclusion rather than to establish formal etiologic diagnoses for each admission.
