# Supplementary material for: Short-term risk stratification using parallel admission and reassessment features in PICU patients with infection
Source: Front Pediatr. 2026 Jun 4;14:1834603. doi: 10.3389/fped.2026.1834603 (PMC13295176; doi:10.3389/fped.2026.1834603)
Supplement: Supplementary file 2 [file Table2.docx]

Supplementary Table S2. Individual-level observed outcomes and predicted probabilities of the four candidate models in the internal test set.

| y | p_lasso | p_xgb | p_rf | p_stack |
| --- | --- | --- | --- | --- |
| 0 | 0.82315 | 0.71575 | 0.56345 | 0.47416 |
| 1 | 0.77756 | 0.76298 | 0.8201 | 0.87562 |
| 0 | 0.18058 | 0.25963 | 0.2951 | 0.07851 |
| 0 | 0.73816 | 0.63025 | 0.58717 | 0.57926 |
| 0 | 0.35082 | 0.31214 | 0.42312 | 0.24017 |
| 1 | 0.37827 | 0.75066 | 0.69637 | 0.73683 |
| 1 | 0.55503 | 0.6882 | 0.70353 | 0.71955 |
| 1 | 0.65047 | 0.94639 | 0.95924 | 0.82551 |
| 1 | 0.88857 | 0.92919 | 0.92511 | 0.87047 |
| 0 | 0.28577 | 0.05455 | 0.13818 | 0.14955 |
| 1 | 0.49542 | 0.36292 | 0.68816 | 0.56399 |
| 1 | 0.09885 | 0.53098 | 0.4651 | 0.41954 |
| 1 | 0.81002 | 0.80526 | 0.72817 | 0.75131 |
| 0 | 0.18408 | 0.25949 | 0.52814 | 0.48128 |
| 1 | 0.2605 | 0.19737 | 0.41187 | 0.6955 |
| 0 | 0.48066 | 0.20557 | 0.61274 | 0.43689 |
| 1 | 0.83666 | 0.6513 | 0.78689 | 0.66491 |
| 0 | 0.43284 | 0.44143 | 0.33314 | 0.37109 |
| 0 | 0.55763 | 0.26111 | 0.32607 | 0.19612 |
| 1 | 0.84246 | 0.71342 | 0.94307 | 0.60317 |
| 1 | 0.74078 | 0.88677 | 0.79632 | 0.73511 |
| 0 | 0.51992 | 0.63371 | 0.75926 | 0.72772 |
| 1 | 0.77666 | 0.50135 | 0.40307 | 0.67972 |
| 1 | 0.6924 | 0.95274 | 0.95642 | 0.94102 |
| 1 | 0.6212 | 0.45847 | 0.65356 | 0.7949 |
| 1 | 0.41547 | 0.24018 | 0.47494 | 0.4905 |
| 0 | 0.33784 | 0.22856 | 0.43164 | 0.26739 |
| 0 | 0.09197 | 0.31 | 0.23073 | 0.17814 |
| 0 | 0.80073 | 0.853 | 0.52946 | 0.47127 |
| 1 | 0.43287 | 0.42931 | 0.27976 | 0.18729 |
| 1 | 0.34705 | 0.74225 | 0.81368 | 0.74015 |
| 1 | 0.90255 | 0.73534 | 0.84926 | 0.76686 |
| 1 | 0.7151 | 0.91626 | 0.95396 | 0.86823 |
| 1 | 0.55709 | 0.84796 | 0.73148 | 0.8133 |
| 0 | 0.27616 | 0.41232 | 0.18966 | 0.13584 |
| 0 | 0.22299 | 0.09094 | 0.23797 | 0.11129 |
| 1 | 0.48958 | 0.83446 | 0.67907 | 0.68226 |
| 1 | 0.74546 | 0.38234 | 0.5574 | 0.72176 |
| 1 | 0.78621 | 0.66222 | 0.60651 | 0.68233 |
| 1 | 0.62423 | 0.644 | 0.73885 | 0.39653 |
| 1 | 0.65878 | 0.67006 | 0.83753 | 0.80414 |
| 0 | 0.46484 | 0.25145 | 0.60817 | 0.50772 |
| 0 | 0.55737 | 0.35058 | 0.21825 | 0.61256 |
| 0 | 0.52926 | 0.24724 | 0.23425 | 0.36243 |
| 1 | 0.32072 | 0.10532 | 0.17974 | 0.27024 |
| 1 | 0.42615 | 0.5225 | 0.50396 | 0.73834 |
| 1 | 0.34171 | 0.66218 | 0.25782 | 0.34927 |
| 1 | 0.64008 | 0.6975 | 0.76288 | 0.69935 |
| 0 | 0.18031 | 0.19209 | 0.17628 | 0.38795 |
| 1 | 0.86353 | 0.79971 | 0.94591 | 0.72782 |
| 1 | 0.659 | 0.83577 | 0.9616 | 0.84525 |
| 1 | 0.25223 | 0.79151 | 0.90041 | 0.7935 |
| 0 | 0.56938 | 0.14736 | 0.42955 | 0.53413 |
| 1 | 0.45842 | 0.42139 | 0.76251 | 0.32372 |
| 1 | 0.38156 | 0.4067 | 0.5287 | 0.48769 |
| 1 | 0.56781 | 0.37121 | 0.57233 | 0.65164 |
| 1 | 0.8321 | 0.50273 | 0.75162 | 0.75946 |
| 0 | 0.30014 | 0.24327 | 0.51939 | 0.44852 |
| 0 | 0.84762 | 0.81872 | 0.92861 | 0.94667 |
| 1 | 0.85696 | 0.9577 | 0.97919 | 0.92262 |
| 1 | 0.82101 | 0.59597 | 0.61837 | 0.59713 |
| 1 | 0.62125 | 0.30136 | 0.36958 | 0.52354 |
| 0 | 0.23057 | 0.31672 | 0.12036 | 0.17049 |
| 0 | 0.17692 | 0.03711 | 0.1003 | 0.11172 |
| 1 | 0.89392 | 0.78015 | 0.88353 | 0.73425 |
| 1 | 0.6943 | 0.84804 | 0.71273 | 0.77134 |
| 0 | 0.26248 | 0.12223 | 0.2432 | 0.31615 |
| 1 | 0.75212 | 0.98154 | 0.90137 | 0.97303 |
| 0 | 0.28725 | 0.65943 | 0.46585 | 0.21704 |
| 0 | 0.34236 | 0.21503 | 0.35785 | 0.19126 |
| 1 | 0.71232 | 0.73246 | 0.88373 | 0.94683 |
| 0 | 0.46081 | 0.70934 | 0.40972 | 0.53019 |
| 0 | 0.60223 | 0.64577 | 0.80825 | 0.74884 |
| 0 | 0.42338 | 0.18139 | 0.49976 | 0.21724 |
| 0 | 0.69801 | 0.84691 | 0.83296 | 0.64846 |
| 0 | 0.56968 | 0.14542 | 0.5983 | 0.29014 |
| 1 | 0.74071 | 0.57104 | 0.48845 | 0.76866 |
| 0 | 0.81078 | 0.87833 | 0.87941 | 0.87018 |
| 1 | 0.51533 | 0.35998 | 0.47292 | 0.47602 |
| 1 | 0.77754 | 0.96751 | 0.50952 | 0.69715 |
| 0 | 0.74933 | 0.09722 | 0.48108 | 0.74346 |
| 1 | 0.69158 | 0.91941 | 0.74358 | 0.81159 |
| 0 | 0.2143 | 0.1898 | 0.14984 | 0.23929 |
| 0 | 0.32277 | 0.24774 | 0.57713 | 0.53561 |
| 1 | 0.93954 | 0.33033 | 0.47561 | 0.80692 |
| 0 | 0.49005 | 0.17958 | 0.1269 | 0.58542 |
| 1 | 0.47205 | 0.7172 | 0.67119 | 0.27764 |
| 0 | 0.55652 | 0.59005 | 0.45937 | 0.4493 |
| 0 | 0.60716 | 0.29629 | 0.53464 | 0.22047 |
| 0 | 0.86438 | 0.70834 | 0.81297 | 0.95182 |
| 0 | 0.06519 | 0.26666 | 0.06684 | 0.49182 |
| 1 | 0.32464 | 0.59687 | 0.14647 | 0.25598 |
| 1 | 0.94853 | 0.94571 | 0.86786 | 0.80237 |
| 1 | 0.89217 | 0.87173 | 0.92755 | 0.82089 |
| 1 | 0.53514 | 0.53208 | 0.7568 | 0.57132 |
| 1 | 0.84326 | 0.73382 | 0.76374 | 0.88503 |
| 1 | 0.5844 | 0.66603 | 0.3603 | 0.45076 |
| 1 | 0.44921 | 0.86591 | 0.92334 | 0.64254 |
| 0 | 0.87022 | 0.76545 | 0.75336 | 0.79567 |
| 0 | 0.89667 | 0.83627 | 0.90132 | 0.75582 |
| 0 | 0.5532 | 0.77948 | 0.6749 | 0.77648 |
| 0 | 0.82241 | 0.31036 | 0.60497 | 0.83658 |
| 0 | 0.74879 | 0.82523 | 0.77358 | 0.51802 |
| 1 | 0.37255 | 0.1931 | 0.5857 | 0.40767 |
| 1 | 0.16908 | 0.5387 | 0.62508 | 0.47584 |
| 1 | 0.78193 | 0.81392 | 0.78931 | 0.8564 |
| 1 | 0.87531 | 0.9304 | 0.87918 | 0.90484 |
| 0 | 0.62485 | 0.48801 | 0.48867 | 0.14036 |
| 1 | 0.96994 | 0.92387 | 0.92962 | 0.9163 |
| 1 | 0.71191 | 0.81319 | 0.87029 | 0.69374 |
| 0 | 0.11937 | 0.20712 | 0.07975 | 0.28978 |
| 0 | 0.22552 | 0.26871 | 0.36832 | 0.22542 |
| 1 | 0.44569 | 0.51304 | 0.63454 | 0.44051 |
| 1 | 0.60838 | 0.48321 | 0.47142 | 0.58201 |
| 1 | 0.29937 | 0.15659 | 0.28632 | 0.28531 |
| 1 | 0.68276 | 0.66205 | 0.81842 | 0.56975 |
| 0 | 0.24056 | 0.286 | 0.46838 | 0.48748 |
| 1 | 0.62301 | 0.71411 | 0.66243 | 0.65016 |
| 1 | 0.90024 | 0.93781 | 0.89907 | 0.93556 |
| 0 | 0.66116 | 0.5598 | 0.57963 | 0.48081 |
| 0 | 0.46272 | 0.17828 | 0.41835 | 0.32795 |
| 0 | 0.27128 | 0.40797 | 0.17975 | 0.23402 |
| 1 | 0.83553 | 0.88457 | 0.9681 | 0.95641 |
| 1 | 0.47889 | 0.71975 | 0.55549 | 0.51293 |
| 1 | 0.14101 | 0.49939 | 0.35717 | 0.56416 |
| 1 | 0.72199 | 0.93977 | 0.91577 | 0.90354 |
| 0 | 0.64441 | 0.69982 | 0.82943 | 0.78255 |
| 0 | 0.45527 | 0.1927 | 0.11592 | 0.12429 |
| 0 | 0.68575 | 0.20408 | 0.44108 | 0.26385 |
| 0 | 0.63143 | 0.53139 | 0.56892 | 0.53899 |
| 1 | 0.89738 | 0.68443 | 0.67942 | 0.59806 |
| 0 | 0.31298 | 0.61254 | 0.35934 | 0.16864 |
| 0 | 0.31307 | 0.2766 | 0.32689 | 0.59048 |
| 1 | 0.48788 | 0.71781 | 0.59016 | 0.57685 |
| 1 | 0.6238 | 0.7235 | 0.50405 | 0.39938 |
| 1 | 0.67762 | 0.77607 | 0.88755 | 0.79637 |
| 0 | 0.40508 | 0.47118 | 0.31295 | 0.28308 |
| 0 | 0.47936 | 0.50226 | 0.51763 | 0.47245 |
| 1 | 0.87234 | 0.57861 | 0.91044 | 0.90733 |
| 1 | 0.75991 | 0.93281 | 0.93425 | 0.86092 |
| 0 | 0.09953 | 0.21783 | 0.18801 | 0.18071 |
| 0 | 0.27024 | 0.68518 | 0.79233 | 0.64311 |
| 1 | 0.29285 | 0.13085 | 0.12202 | 0.22528 |
| 0 | 0.22607 | 0.06636 | 0.12355 | 0.16423 |
| 1 | 0.58342 | 0.76016 | 0.8539 | 0.80886 |
| 1 | 0.07597 | 0.52748 | 0.11331 | 0.1474 |
| 1 | 0.42264 | 0.32321 | 0.41526 | 0.40623 |
| 0 | 0.58686 | 0.88645 | 0.65679 | 0.65784 |
| 0 | 0.66983 | 0.37498 | 0.47638 | 0.53762 |
| 0 | 0.12229 | 0.19524 | 0.17254 | 0.31799 |
| 0 | 0.58182 | 0.49425 | 0.54024 | 0.48697 |
| 1 | 0.65484 | 0.80699 | 0.53861 | 0.47189 |
| 1 | 0.90029 | 0.74429 | 0.77177 | 0.65923 |
| 1 | 0.78247 | 0.67246 | 0.93101 | 0.56871 |
| 1 | 0.54881 | 0.68236 | 0.72937 | 0.54449 |
| 0 | 0.54759 | 0.40422 | 0.28691 | 0.22675 |
| 1 | 0.37291 | 0.13319 | 0.32894 | 0.55933 |
| 1 | 0.61557 | 0.74746 | 0.57967 | 0.70105 |
| 1 | 0.45449 | 0.84538 | 0.38754 | 0.7158 |
| 0 | 0.38108 | 0.3298 | 0.10062 | 0.16134 |
| 0 | 0.47349 | 0.24892 | 0.41106 | 0.38411 |
| 0 | 0.11954 | 0.20225 | 0.14158 | 0.22984 |
| 1 | 0.3843 | 0.55216 | 0.6258 | 0.48436 |
| 1 | 0.88705 | 0.86416 | 0.96258 | 0.9668 |
| 1 | 0.83269 | 0.96918 | 0.9892 | 0.94018 |
| 0 | 0.18646 | 0.61764 | 0.68328 | 0.71678 |
| 1 | 0.90776 | 0.87584 | 0.95914 | 0.81164 |
| 1 | 0.86819 | 0.54663 | 0.6225 | 0.73665 |
| 1 | 0.47012 | 0.13836 | 0.24873 | 0.08237 |
| 0 | 0.77141 | 0.90098 | 0.94797 | 0.95466 |
| 1 | 0.58706 | 0.61033 | 0.49113 | 0.53273 |
| 0 | 0.81401 | 0.46152 | 0.67425 | 0.75127 |
| 0 | 0.53736 | 0.46785 | 0.66622 | 0.43656 |
| 1 | 0.94814 | 0.88734 | 0.90354 | 0.96314 |
| 0 | 0.51605 | 0.27578 | 0.09144 | 0.30959 |
| 1 | 0.41566 | 0.60815 | 0.61351 | 0.62728 |
| 1 | 0.75929 | 0.60542 | 0.83585 | 0.68582 |
| 1 | 0.11869 | 0.7097 | 0.82396 | 0.608 |
| 0 | 0.88924 | 0.28899 | 0.33138 | 0.34558 |
| 0 | 0.5226 | 0.6836 | 0.6566 | 0.61107 |
| 1 | 0.41287 | 0.91458 | 0.8686 | 0.88512 |
| 1 | 0.69961 | 0.63983 | 0.63426 | 0.60342 |
| 0 | 0.46436 | 0.51774 | 0.22992 | 0.25836 |
| 0 | 0.61994 | 0.26939 | 0.69556 | 0.38362 |
| 1 | 0.24664 | 0.62976 | 0.464 | 0.44398 |
| 1 | 0.779 | 0.73407 | 0.9004 | 0.76941 |
| 1 | 0.09568 | 0.4935 | 0.42004 | 0.25538 |
| 0 | 0.44088 | 0.21489 | 0.23854 | 0.19531 |
| 1 | 0.49797 | 0.76418 | 0.63221 | 0.40913 |
| 0 | 0.27068 | 0.48607 | 0.2976 | 0.19933 |
| 1 | 0.83353 | 0.67687 | 0.81441 | 0.71816 |
| 1 | 0.43683 | 0.92857 | 0.86101 | 0.73631 |
| 0 | 0.2706 | 0.46753 | 0.24534 | 0.22154 |
| 0 | 0.24928 | 0.57398 | 0.13776 | 0.23179 |
| 0 | 0.15176 | 0.23143 | 0.108 | 0.24445 |
| 1 | 0.79997 | 0.88284 | 0.62372 | 0.68264 |
| 0 | 0.50208 | 0.4373 | 0.19568 | 0.30973 |
| 0 | 0.33219 | 0.36126 | 0.4076 | 0.28435 |
| 1 | 0.67148 | 0.93939 | 0.80162 | 0.64977 |
| 1 | 0.58926 | 0.28479 | 0.61011 | 0.73362 |
| 0 | 0.60983 | 0.73405 | 0.49848 | 0.54022 |
| 0 | 0.47788 | 0.52863 | 0.51519 | 0.63782 |
| 1 | 0.51432 | 0.68716 | 0.52773 | 0.39398 |
| 1 | 0.44809 | 0.85313 | 0.85433 | 0.81753 |
| 1 | 0.59706 | 0.90064 | 0.88872 | 0.81106 |
| 1 | 0.38185 | 0.67462 | 0.45463 | 0.4512 |
| 1 | 0.63185 | 0.79955 | 0.91063 | 0.79121 |
| 1 | 0.51905 | 0.11384 | 0.39652 | 0.40899 |
| 0 | 0.6476 | 0.7172 | 0.83167 | 0.72752 |
| 0 | 0.53144 | 0.54395 | 0.32554 | 0.33268 |
| 0 | 0.18231 | 0.35243 | 0.28359 | 0.24354 |
| 1 | 0.65206 | 0.5506 | 0.51401 | 0.49756 |
| 1 | 0.55734 | 0.75033 | 0.88733 | 0.55361 |
| 0 | 0.29152 | 0.53377 | 0.31387 | 0.39417 |
| 1 | 0.56583 | 0.66801 | 0.6886 | 0.71558 |
| 1 | 0.74386 | 0.55978 | 0.63197 | 0.6544 |
| 1 | 0.56488 | 0.21858 | 0.12678 | 0.21606 |
| 1 | 0.08977 | 0.21509 | 0.14061 | 0.12115 |
| 1 | 0.88743 | 0.77399 | 0.84824 | 0.68088 |
| 0 | 0.30022 | 0.37496 | 0.85783 | 0.7586 |
| 1 | 0.41405 | 0.24233 | 0.10844 | 0.1014 |
| 0 | 0.48469 | 0.78933 | 0.18282 | 0.28781 |
| 0 | 0.42445 | 0.23663 | 0.68853 | 0.52258 |
| 1 | 0.59472 | 0.86453 | 0.8268 | 0.84962 |
| 1 | 0.53502 | 0.71437 | 0.57767 | 0.51566 |
| 0 | 0.67169 | 0.73804 | 0.89317 | 0.7269 |
| 1 | 0.58157 | 0.68914 | 0.75155 | 0.8291 |
| 0 | 0.22421 | 0.32975 | 0.19848 | 0.30953 |
| 1 | 0.91479 | 0.92425 | 0.91595 | 0.80501 |
| 0 | 0.6531 | 0.74085 | 0.69587 | 0.60061 |
| 1 | 0.77645 | 0.72253 | 0.35241 | 0.72392 |
| 1 | 0.82145 | 0.79482 | 0.9045 | 0.73788 |
| 1 | 0.62112 | 0.7955 | 0.72184 | 0.80813 |
| 0 | 0.50675 | 0.50506 | 0.45375 | 0.69196 |
| 0 | 0.33518 | 0.25993 | 0.07277 | 0.13457 |
| 0 | 0.62584 | 0.41453 | 0.42145 | 0.35819 |
| 0 | 0.36512 | 0.77387 | 0.74798 | 0.66264 |
| 0 | 0.58151 | 0.3932 | 0.33535 | 0.30236 |
| 0 | 0.71224 | 0.65638 | 0.83096 | 0.80739 |
| 1 | 0.64466 | 0.74249 | 0.51341 | 0.62229 |
| 0 | 0.45483 | 0.20448 | 0.50458 | 0.18857 |
| 1 | 0.63143 | 0.86449 | 0.66227 | 0.77346 |
| 1 | 0.47783 | 0.25194 | 0.20163 | 0.36447 |
| 1 | 0.39539 | 0.17314 | 0.40318 | 0.64204 |
| 1 | 0.24792 | 0.83452 | 0.70094 | 0.4247 |
| 0 | 0.77531 | 0.56437 | 0.18782 | 0.16043 |
| 0 | 0.37273 | 0.48571 | 0.39576 | 0.55105 |
| 1 | 0.41051 | 0.31084 | 0.35081 | 0.7403 |
| 1 | 0.89211 | 0.70113 | 0.8709 | 0.85398 |
| 1 | 0.31432 | 0.56836 | 0.37658 | 0.28507 |
| 1 | 0.60507 | 0.59027 | 0.84734 | 0.654 |
| 0 | 0.64054 | 0.71085 | 0.59699 | 0.6614 |
| 1 | 0.4427 | 0.34127 | 0.54003 | 0.34704 |
| 0 | 0.46659 | 0.5966 | 0.72764 | 0.593 |
| 1 | 0.48244 | 0.26612 | 0.56472 | 0.60579 |
| 1 | 0.31423 | 0.19115 | 0.15881 | 0.06834 |
| 1 | 0.18474 | 0.12397 | 0.1765 | 0.11985 |
| 0 | 0.1458 | 0.24548 | 0.08114 | 0.22516 |
| 0 | 0.26734 | 0.34186 | 0.3721 | 0.52494 |
| 1 | 0.82631 | 0.68239 | 0.5651 | 0.48145 |
| 0 | 0.19336 | 0.51807 | 0.13845 | 0.1397 |
| 1 | 0.41768 | 0.37522 | 0.40935 | 0.34505 |
| 1 | 0.58617 | 0.41792 | 0.52267 | 0.51394 |
| 0 | 0.77268 | 0.73426 | 0.83113 | 0.46881 |
| 0 | 0.37915 | 0.10859 | 0.65426 | 0.11893 |
| 0 | 0.13493 | 0.13218 | 0.19589 | 0.11609 |
| 1 | 0.38303 | 0.55825 | 0.42913 | 0.26691 |
| 1 | 0.67874 | 0.5685 | 0.8711 | 0.62917 |
| 0 | 0.23671 | 0.24034 | 0.36004 | 0.68296 |
| 1 | 0.44759 | 0.43609 | 0.37965 | 0.21024 |
| 1 | 0.64182 | 0.63515 | 0.72252 | 0.80432 |
| 0 | 0.62604 | 0.48149 | 0.41081 | 0.51721 |
| 1 | 0.92503 | 0.84177 | 0.95434 | 0.9665 |
| 0 | 0.83777 | 0.65107 | 0.73271 | 0.7571 |
| 1 | 0.70459 | 0.55301 | 0.7577 | 0.61138 |
| 0 | 0.13373 | 0.15762 | 0.03031 | 0.15238 |
| 1 | 0.16771 | 0.13805 | 0.06536 | 0.07387 |
| 0 | 0.50482 | 0.67547 | 0.61314 | 0.66795 |
| 0 | 0.35656 | 0.67889 | 0.59307 | 0.58104 |
| 0 | 0.08235 | 0.13315 | 0.03088 | 0.01626 |
| 0 | 0.43356 | 0.70491 | 0.73876 | 0.61261 |
| 1 | 0.90421 | 0.53203 | 0.94338 | 0.70595 |
| 0 | 0.46684 | 0.78374 | 0.80725 | 0.86569 |
| 0 | 0.33051 | 0.61485 | 0.68982 | 0.37145 |
| 1 | 0.8306 | 0.91503 | 0.87999 | 0.92206 |
| 1 | 0.61529 | 0.56119 | 0.72043 | 0.81494 |
| 1 | 0.93965 | 0.89732 | 0.92649 | 0.84274 |
| 0 | 0.81834 | 0.54762 | 0.74848 | 0.65773 |
| 0 | 0.67419 | 0.59064 | 0.34672 | 0.61438 |
| 1 | 0.33024 | 0.11474 | 0.15743 | 0.09984 |
| 1 | 0.59491 | 0.38967 | 0.68638 | 0.35832 |
| 0 | 0.21921 | 0.19145 | 0.09012 | 0.22004 |
| 1 | 0.90981 | 0.63782 | 0.6269 | 0.71883 |
| 1 | 0.62958 | 0.26224 | 0.59938 | 0.37782 |
| 0 | 0.25894 | 0.57737 | 0.69941 | 0.82321 |
| 0 | 0.35103 | 0.56996 | 0.51758 | 0.28672 |
| 0 | 0.39282 | 0.84545 | 0.68683 | 0.66082 |
| 0 | 0.52835 | 0.33583 | 0.14994 | 0.31733 |
| 0 | 0.49416 | 0.53897 | 0.12301 | 0.11698 |
| 1 | 0.05584 | 0.71613 | 0.36397 | 0.249 |
| 1 | 0.94104 | 0.94198 | 0.91765 | 0.91781 |
| 1 | 0.5848 | 0.66183 | 0.87799 | 0.68559 |
| 0 | 0.48052 | 0.10002 | 0.08393 | 0.11558 |
| 1 | 0.62149 | 0.52139 | 0.61716 | 0.28242 |
| 1 | 0.23766 | 0.30888 | 0.43859 | 0.57011 |
| 1 | 0.59771 | 0.55344 | 0.4995 | 0.16009 |
| 0 | 0.36836 | 0.23963 | 0.22027 | 0.38216 |
| 1 | 0.30855 | 0.63336 | 0.45596 | 0.39163 |
| 1 | 0.58548 | 0.70567 | 0.80561 | 0.64841 |
| 0 | 0.17385 | 0.3143 | 0.18287 | 0.08093 |
| 1 | 0.46519 | 0.29398 | 0.72272 | 0.40143 |
| 0 | 0.05939 | 0.02588 | 0.07258 | 0.06635 |
| 1 | 0.58154 | 0.70805 | 0.55161 | 0.77765 |
| 0 | 0.14496 | 0.27612 | 0.48557 | 0.6189 |
| 0 | 0.76148 | 0.82537 | 0.69694 | 0.52664 |
| 1 | 0.81946 | 0.67448 | 0.47757 | 0.52929 |
| 0 | 0.63998 | 0.67476 | 0.60698 | 0.50419 |
| 0 | 0.1831 | 0.16243 | 0.04594 | 0.31241 |
| 0 | 0.59704 | 0.85854 | 0.40417 | 0.55826 |
| 1 | 0.62398 | 0.74712 | 0.78113 | 0.8946 |
| 0 | 0.40008 | 0.08248 | 0.09561 | 0.09665 |
| 0 | 0.29557 | 0.51501 | 0.44632 | 0.23291 |
| 1 | 0.46108 | 0.2147 | 0.59228 | 0.57312 |
| 0 | 0.63807 | 0.60727 | 0.57319 | 0.55122 |
| 1 | 0.56617 | 0.6902 | 0.85522 | 0.74736 |
| 0 | 0.42867 | 0.3366 | 0.62459 | 0.74106 |
| 0 | 0.0795 | 0.12048 | 0.03392 | 0.22924 |
| 0 | 0.19263 | 0.20704 | 0.13308 | 0.23691 |
| 1 | 0.70998 | 0.83123 | 0.58316 | 0.57957 |
| 1 | 0.81159 | 0.94018 | 0.90951 | 0.91163 |
| 1 | 0.79652 | 0.63693 | 0.24259 | 0.58664 |
| 1 | 0.65536 | 0.41925 | 0.74263 | 0.64765 |
| 0 | 0.66131 | 0.28006 | 0.4031 | 0.57416 |
| 0 | 0.12661 | 0.13957 | 0.04604 | 0.06897 |
| 1 | 0.57102 | 0.69808 | 0.46675 | 0.43061 |
| 1 | 0.19211 | 0.54263 | 0.56749 | 0.573 |
| 1 | 0.36748 | 0.50966 | 0.53829 | 0.68774 |
| 1 | 0.74636 | 0.59038 | 0.46682 | 0.53198 |
| 1 | 0.46108 | 0.64694 | 0.7359 | 0.32644 |
| 0 | 0.57865 | 0.341 | 0.27607 | 0.32723 |
| 1 | 0.20956 | 0.24321 | 0.56899 | 0.41142 |
| 0 | 0.76871 | 0.32245 | 0.81135 | 0.73108 |
| 1 | 0.44861 | 0.83356 | 0.64631 | 0.6741 |
| 1 | 0.29416 | 0.08904 | 0.52594 | 0.23642 |
| 1 | 0.79661 | 0.46867 | 0.70207 | 0.38578 |
| 1 | 0.585 | 0.42833 | 0.44987 | 0.73452 |
| 0 | 0.22973 | 0.15125 | 0.20484 | 0.22481 |
| 0 | 0.70095 | 0.88934 | 0.75811 | 0.54513 |
| 0 | 0.7861 | 0.64673 | 0.89717 | 0.88577 |
| 0 | 0.34419 | 0.87653 | 0.67356 | 0.27977 |
| 1 | 0.54553 | 0.8824 | 0.69143 | 0.74238 |
| 1 | 0.51791 | 0.92106 | 0.72279 | 0.69751 |
| 1 | 0.16501 | 0.40677 | 0.33259 | 0.25241 |
| 0 | 0.28975 | 0.23996 | 0.50233 | 0.32764 |
| 0 | 0.86189 | 0.4015 | 0.6811 | 0.62563 |
| 1 | 0.4351 | 0.15758 | 0.24198 | 0.67155 |
| 1 | 0.34388 | 0.324 | 0.56179 | 0.52828 |
| 1 | 0.91526 | 0.64168 | 0.80295 | 0.69984 |
| 1 | 0.65334 | 0.5181 | 0.74161 | 0.60296 |
| 0 | 0.48286 | 0.4443 | 0.6695 | 0.43614 |
| 1 | 0.3135 | 0.27633 | 0.56251 | 0.09997 |
| 1 | 0.70359 | 0.7025 | 0.81446 | 0.49277 |
| 1 | 0.09806 | 0.18407 | 0.10837 | 0.06037 |
| 1 | 0.70876 | 0.29127 | 0.61032 | 0.37287 |
| 0 | 0.59704 | 0.56518 | 0.41824 | 0.34564 |
| 0 | 0.52836 | 0.45399 | 0.15177 | 0.17681 |
| 0 | 0.48394 | 0.10679 | 0.32173 | 0.46792 |
| 1 | 0.47766 | 0.69361 | 0.30739 | 0.64499 |
| 1 | 0.77133 | 0.83243 | 0.85185 | 0.56258 |
| 0 | 0.39047 | 0.68837 | 0.32651 | 0.51598 |
| 0 | 0.21175 | 0.32001 | 0.48697 | 0.48155 |
| 0 | 0.40631 | 0.40355 | 0.3328 | 0.29372 |
| 0 | 0.22578 | 0.63229 | 0.46782 | 0.21154 |
| 1 | 0.82893 | 0.74963 | 0.91653 | 0.9091 |
| 1 | 0.66007 | 0.69707 | 0.71226 | 0.78022 |
| 1 | 0.16484 | 0.6083 | 0.833 | 0.76635 |
| 0 | 0.79401 | 0.55193 | 0.42882 | 0.38397 |
| 0 | 0.36808 | 0.17542 | 0.0518 | 0.0797 |
| 1 | 0.57595 | 0.79409 | 0.92045 | 0.7962 |
| 1 | 0.60802 | 0.80191 | 0.57825 | 0.88012 |
| 1 | 0.80337 | 0.65131 | 0.96686 | 0.82938 |
| 1 | 0.53646 | 0.45552 | 0.76249 | 0.75288 |
| 0 | 0.40157 | 0.63335 | 0.6459 | 0.57644 |
| 1 | 0.51652 | 0.50822 | 0.5698 | 0.5855 |
| 0 | 0.16145 | 0.37654 | 0.29248 | 0.53553 |
| 0 | 0.09232 | 0.07557 | 0.12514 | 0.18379 |
| 1 | 0.34457 | 0.51216 | 0.73596 | 0.53551 |
| 0 | 0.93579 | 0.8474 | 0.83781 | 0.91298 |
| 1 | 0.82247 | 0.90851 | 0.83028 | 0.87509 |
| 0 | 0.41279 | 0.17284 | 0.16272 | 0.3283 |
| 1 | 0.77017 | 0.78654 | 0.81824 | 0.89899 |
| 0 | 0.72396 | 0.70153 | 0.58848 | 0.54724 |
| 1 | 0.48088 | 0.48945 | 0.38311 | 0.23996 |
| 0 | 0.63412 | 0.56764 | 0.6627 | 0.91155 |
| 1 | 0.52583 | 0.55362 | 0.79889 | 0.72125 |
| 1 | 0.19937 | 0.52075 | 0.1363 | 0.29745 |
| 1 | 0.61515 | 0.59482 | 0.65873 | 0.50497 |
| 0 | 0.05939 | 0.39303 | 0.07204 | 0.18158 |
| 1 | 0.65813 | 0.63281 | 0.78091 | 0.41218 |
| 1 | 0.68539 | 0.53277 | 0.44606 | 0.47625 |
| 1 | 0.88122 | 0.96092 | 0.89778 | 0.97398 |
| 1 | 0.95634 | 0.9526 | 0.97348 | 0.97186 |
| 0 | 0.84965 | 0.75295 | 0.81511 | 0.77054 |
| 1 | 0.09982 | 0.67995 | 0.47372 | 0.65888 |
| 1 | 0.77065 | 0.19488 | 0.27864 | 0.39108 |
| 1 | 0.82614 | 0.58206 | 0.82457 | 0.52017 |
| 1 | 0.60925 | 0.80525 | 0.81164 | 0.73284 |
| 0 | 0.32834 | 0.22785 | 0.08857 | 0.10143 |
| 1 | 0.86019 | 0.80035 | 0.9285 | 0.96604 |
| 1 | 0.83713 | 0.47443 | 0.7901 | 0.65814 |
| 1 | 0.18572 | 0.09235 | 0.50701 | 0.16787 |
| 1 | 0.40256 | 0.276 | 0.14186 | 0.21776 |
| 0 | 0.59899 | 0.57088 | 0.92073 | 0.75377 |
| 1 | 0.83508 | 0.67609 | 0.62776 | 0.957 |
| 1 | 0.19061 | 0.82698 | 0.7099 | 0.81387 |
| 1 | 0.52856 | 0.61658 | 0.73292 | 0.68908 |
| 1 | 0.16382 | 0.06622 | 0.21685 | 0.12646 |
| 1 | 0.41407 | 0.42287 | 0.74199 | 0.68769 |
| 0 | 0.43575 | 0.6397 | 0.77734 | 0.49523 |
| 1 | 0.80065 | 0.8729 | 0.94585 | 0.85365 |
| 1 | 0.70517 | 0.59543 | 0.82468 | 0.37612 |
| 0 | 0.23134 | 0.2154 | 0.28095 | 0.23168 |
| 1 | 0.36222 | 0.84974 | 0.41736 | 0.54641 |
| 0 | 0.52322 | 0.42198 | 0.51789 | 0.44401 |
| 0 | 0.34864 | 0.09867 | 0.12187 | 0.09761 |
| 1 | 0.40453 | 0.30483 | 0.48941 | 0.45813 |
| 1 | 0.80804 | 0.96346 | 0.91448 | 0.93651 |
| 1 | 0.7337 | 0.83154 | 0.85766 | 0.72763 |
| 1 | 0.91562 | 0.44564 | 0.8953 | 0.38279 |
| 0 | 0.22478 | 0.05183 | 0.11468 | 0.1092 |
| 1 | 0.33782 | 0.71063 | 0.5019 | 0.68986 |
| 1 | 0.87543 | 0.68124 | 0.59977 | 0.55029 |
| 1 | 0.54798 | 0.35499 | 0.64285 | 0.26898 |
| 1 | 0.73438 | 0.96256 | 0.89261 | 0.80277 |
| 1 | 0.76714 | 0.92828 | 0.747 | 0.71814 |
| 1 | 0.56708 | 0.40347 | 0.6582 | 0.48441 |
| 1 | 0.39374 | 0.72372 | 0.71016 | 0.61067 |
| 0 | 0.43951 | 0.44728 | 0.70802 | 0.73037 |
| 0 | 0.59487 | 0.95699 | 0.8055 | 0.6085 |
| 1 | 0.26049 | 0.68069 | 0.51673 | 0.56101 |
| 0 | 0.14726 | 0.13462 | 0.13493 | 0.25551 |
| 1 | 0.68615 | 0.82113 | 0.9458 | 0.85223 |
| 0 | 0.6035 | 0.45494 | 0.56555 | 0.55221 |
| 1 | 0.32986 | 0.47485 | 0.25855 | 0.14536 |
| 1 | 0.47163 | 0.48992 | 0.326 | 0.43987 |
| 1 | 0.5926 | 0.09207 | 0.32127 | 0.51084 |
| 1 | 0.21448 | 0.41239 | 0.17605 | 0.31268 |
| 0 | 0.76451 | 0.72774 | 0.85854 | 0.77917 |
| 0 | 0.13791 | 0.18786 | 0.08575 | 0.17969 |
| 1 | 0.43815 | 0.56306 | 0.52116 | 0.32073 |
| 1 | 0.26879 | 0.85281 | 0.86441 | 0.76405 |
| 1 | 0.79587 | 0.38261 | 0.15885 | 0.50464 |
| 1 | 0.82514 | 0.73897 | 0.64348 | 0.45734 |
| 1 | 0.33762 | 0.25579 | 0.44212 | 0.44849 |
| 0 | 0.15831 | 0.13418 | 0.22216 | 0.18399 |
| 1 | 0.44735 | 0.54309 | 0.76908 | 0.29676 |
| 1 | 0.21498 | 0.72367 | 0.44042 | 0.29779 |
| 1 | 0.73692 | 0.86633 | 0.78244 | 0.79136 |
| 0 | 0.51601 | 0.37174 | 0.51761 | 0.8366 |
| 1 | 0.44895 | 0.68856 | 0.55203 | 0.7023 |
| 1 | 0.52328 | 0.503 | 0.81822 | 0.74432 |
| 1 | 0.24849 | 0.62089 | 0.70665 | 0.68012 |
| 0 | 0.10546 | 0.12563 | 0.0362 | 0.06863 |
| 1 | 0.30009 | 0.91361 | 0.7989 | 0.66833 |
| 0 | 0.24637 | 0.29149 | 0.33699 | 0.12936 |
| 0 | 0.58292 | 0.58319 | 0.76584 | 0.31945 |
| 1 | 0.33745 | 0.15746 | 0.36037 | 0.18295 |
| 1 | 0.69384 | 0.72095 | 0.74781 | 0.67316 |
| 1 | 0.31648 | 0.44156 | 0.10394 | 0.25008 |
| 1 | 0.75619 | 0.44461 | 0.5116 | 0.67939 |
| 1 | 0.7181 | 0.38181 | 0.86687 | 0.92391 |
| 0 | 0.52322 | 0.61014 | 0.81432 | 0.67528 |
| 0 | 0.27039 | 0.72481 | 0.55471 | 0.44659 |
| 0 | 0.27594 | 0.17717 | 0.1326 | 0.29261 |
| 0 | 0.66886 | 0.45499 | 0.31758 | 0.43648 |
| 0 | 0.63863 | 0.30134 | 0.28288 | 0.13101 |
| 1 | 0.58259 | 0.71562 | 0.83155 | 0.75694 |
| 0 | 0.11136 | 0.25906 | 0.20631 | 0.41576 |
| 0 | 0.27561 | 0.42747 | 0.45841 | 0.33738 |
| 0 | 0.50475 | 0.27758 | 0.12617 | 0.10431 |
| 1 | 0.36458 | 0.2913 | 0.22318 | 0.59853 |
| 0 | 0.16738 | 0.32272 | 0.17095 | 0.20857 |
| 1 | 0.36773 | 0.8467 | 0.40255 | 0.65986 |
| 1 | 0.24504 | 0.68104 | 0.31956 | 0.37486 |
| 1 | 0.66412 | 0.78111 | 0.82258 | 0.84371 |
| 0 | 0.81858 | 0.59858 | 0.55262 | 0.77299 |
| 1 | 0.28224 | 0.4768 | 0.33305 | 0.5442 |
| 1 | 0.62735 | 0.87521 | 0.87003 | 0.70577 |
| 0 | 0.42207 | 0.06264 | 0.10869 | 0.13276 |
| 1 | 0.21975 | 0.20269 | 0.1973 | 0.16016 |
| 0 | 0.32411 | 0.64005 | 0.19049 | 0.40147 |
| 0 | 0.06785 | 0.06065 | 0.15361 | 0.10474 |
| 1 | 0.43983 | 0.43438 | 0.25284 | 0.5413 |
| 0 | 0.48315 | 0.73536 | 0.72909 | 0.53106 |
| 0 | 0.65139 | 0.22001 | 0.25203 | 0.17821 |
| 1 | 0.2837 | 0.51403 | 0.31504 | 0.43012 |
| 1 | 0.61316 | 0.46031 | 0.40274 | 0.41288 |
| 1 | 0.29742 | 0.31327 | 0.24293 | 0.36208 |
| 1 | 0.54157 | 0.3303 | 0.12689 | 0.26272 |
| 1 | 0.67396 | 0.38457 | 0.82354 | 0.8091 |
| 0 | 0.7237 | 0.3989 | 0.5585 | 0.43125 |
| 0 | 0.40953 | 0.55813 | 0.24897 | 0.19402 |
| 1 | 0.68299 | 0.85403 | 0.73111 | 0.69593 |
| 0 | 0.45896 | 0.50122 | 0.2768 | 0.44759 |
| 0 | 0.87094 | 0.50432 | 0.72284 | 0.80531 |
| 1 | 0.46649 | 0.53518 | 0.86686 | 0.69363 |
| 1 | 0.57515 | 0.87113 | 0.89807 | 0.79053 |
| 0 | 0.1408 | 0.37766 | 0.20724 | 0.26794 |
| 0 | 0.3918 | 0.60384 | 0.90556 | 0.91561 |
| 0 | 0.30468 | 0.64908 | 0.43342 | 0.67907 |
| 0 | 0.3697 | 0.44478 | 0.37818 | 0.26646 |
| 1 | 0.75065 | 0.62695 | 0.80954 | 0.73903 |
| 1 | 0.78069 | 0.36733 | 0.38047 | 0.74963 |
| 0 | 0.74506 | 0.30549 | 0.64443 | 0.68668 |
| 1 | 0.85348 | 0.72848 | 0.83213 | 0.65371 |
| 1 | 0.12871 | 0.80103 | 0.72322 | 0.8718 |
| 1 | 0.48492 | 0.28468 | 0.48641 | 0.47352 |
| 1 | 0.57912 | 0.71968 | 0.28309 | 0.50743 |
| 0 | 0.82899 | 0.92028 | 0.85846 | 0.66086 |
| 0 | 0.40584 | 0.10764 | 0.43531 | 0.56865 |
| 1 | 0.61669 | 0.68191 | 0.72517 | 0.72647 |
| 1 | 0.59316 | 0.73108 | 0.65723 | 0.80098 |
| 0 | 0.81266 | 0.13772 | 0.23471 | 0.26988 |
| 1 | 0.71241 | 0.87654 | 0.96012 | 0.94709 |
| 0 | 0.47953 | 0.57534 | 0.47782 | 0.71292 |
| 1 | 0.80935 | 0.61948 | 0.79673 | 0.82111 |
| 1 | 0.9253 | 0.83912 | 0.90626 | 0.94852 |
| 1 | 0.85051 | 0.54086 | 0.65706 | 0.78726 |
| 0 | 0.35353 | 0.75602 | 0.88632 | 0.77347 |
| 1 | 0.38982 | 0.70978 | 0.56002 | 0.6351 |
| 1 | 0.8884 | 0.95449 | 0.43972 | 0.56272 |
| 1 | 0.90642 | 0.93948 | 0.93875 | 0.82535 |
| 0 | 0.27273 | 0.51075 | 0.65524 | 0.7565 |
| 0 | 0.18312 | 0.25666 | 0.28324 | 0.24539 |
| 0 | 0.3222 | 0.20089 | 0.28661 | 0.10382 |
| 1 | 0.74593 | 0.47168 | 0.65645 | 0.65817 |
| 1 | 0.69176 | 0.44362 | 0.52615 | 0.7838 |
| 0 | 0.17936 | 0.39094 | 0.24779 | 0.71366 |
| 0 | 0.67288 | 0.68026 | 0.74907 | 0.7174 |
| 0 | 0.35839 | 0.33125 | 0.2309 | 0.22852 |
| 0 | 0.62572 | 0.4179 | 0.51852 | 0.24051 |
| 1 | 0.56576 | 0.94672 | 0.61248 | 0.72406 |
| 1 | 0.91084 | 0.67371 | 0.81442 | 0.70014 |
| 1 | 0.84325 | 0.56989 | 0.8449 | 0.83271 |
| 1 | 0.77597 | 0.42154 | 0.73591 | 0.87711 |
| 0 | 0.52182 | 0.29651 | 0.54358 | 0.60829 |
| 0 | 0.32682 | 0.42122 | 0.5575 | 0.48792 |
| 1 | 0.5785 | 0.76713 | 0.77288 | 0.87979 |
| 1 | 0.18038 | 0.15342 | 0.16218 | 0.6976 |
| 1 | 0.83968 | 0.66999 | 0.73271 | 0.84356 |
| 1 | 0.45626 | 0.4436 | 0.30201 | 0.756 |
| 0 | 0.55798 | 0.25105 | 0.29893 | 0.3298 |
| 1 | 0.45279 | 0.28648 | 0.61847 | 0.59202 |
| 0 | 0.21054 | 0.49017 | 0.57089 | 0.16405 |
| 0 | 0.45155 | 0.11747 | 0.27995 | 0.20953 |
| 0 | 0.18405 | 0.26048 | 0.03206 | 0.05387 |
| 0 | 0.21377 | 0.2777 | 0.33581 | 0.40332 |
| 0 | 0.88047 | 0.78898 | 0.78413 | 0.77333 |
| 0 | 0.40698 | 0.79512 | 0.87544 | 0.94001 |
| 0 | 0.30126 | 0.84758 | 0.86943 | 0.8805 |
| 0 | 0.72673 | 0.44936 | 0.37492 | 0.17764 |
| 1 | 0.23004 | 0.582 | 0.26473 | 0.33049 |
| 0 | 0.4294 | 0.53056 | 0.31472 | 0.56744 |
| 0 | 0.63147 | 0.65218 | 0.59895 | 0.69365 |
| 1 | 0.8496 | 0.86946 | 0.90901 | 0.8135 |
| 0 | 0.70149 | 0.75049 | 0.79364 | 0.52922 |
| 1 | 0.85865 | 0.39431 | 0.74053 | 0.66708 |
| 0 | 0.39942 | 0.80003 | 0.89364 | 0.82892 |
| 1 | 0.8638 | 0.75648 | 0.68719 | 0.70277 |
| 0 | 0.24329 | 0.31427 | 0.45647 | 0.22722 |
| 1 | 0.70624 | 0.58709 | 0.82782 | 0.9085 |
| 1 | 0.75291 | 0.87574 | 0.91043 | 0.52625 |
| 1 | 0.62934 | 0.67132 | 0.51065 | 0.55756 |
| 1 | 0.56544 | 0.40896 | 0.87673 | 0.79959 |
| 0 | 0.38585 | 0.6336 | 0.636 | 0.6292 |
| 0 | 0.44738 | 0.51592 | 0.37719 | 0.3656 |
| 0 | 0.37084 | 0.09646 | 0.24569 | 0.41701 |
| 0 | 0.69639 | 0.73173 | 0.45795 | 0.62567 |
| 1 | 0.65044 | 0.91225 | 0.83574 | 0.88128 |
| 1 | 0.5989 | 0.52983 | 0.36445 | 0.30141 |
| 1 | 0.43273 | 0.47264 | 0.52292 | 0.44871 |
| 0 | 0.22655 | 0.6426 | 0.58454 | 0.58123 |
| 0 | 0.55734 | 0.67202 | 0.50077 | 0.24871 |
| 1 | 0.93 | 0.63108 | 0.80026 | 0.75248 |
| 1 | 0.86157 | 0.6774 | 0.75022 | 0.91299 |
| 1 | 0.66275 | 0.6752 | 0.62527 | 0.39587 |
| 0 | 0.61547 | 0.79096 | 0.82324 | 0.60539 |
| 0 | 0.49347 | 0.31941 | 0.46576 | 0.32231 |
| 0 | 0.1065 | 0.33028 | 0.09934 | 0.15904 |
| 0 | 0.24211 | 0.32788 | 0.42378 | 0.34199 |
| 0 | 0.10512 | 0.23716 | 0.10683 | 0.16876 |
| 0 | 0.51162 | 0.07076 | 0.11952 | 0.08262 |
| 1 | 0.41745 | 0.33701 | 0.46826 | 0.57131 |
| 1 | 0.38179 | 0.65844 | 0.64508 | 0.59588 |
| 0 | 0.68969 | 0.29869 | 0.75916 | 0.35112 |
| 0 | 0.7178 | 0.75022 | 0.60081 | 0.65554 |
| 1 | 0.89642 | 0.66694 | 0.935 | 0.89341 |
| 1 | 0.97851 | 0.67392 | 0.92501 | 0.73128 |
| 0 | 0.55037 | 0.38337 | 0.26212 | 0.15415 |
| 1 | 0.55988 | 0.30941 | 0.20758 | 0.32219 |
| 0 | 0.55336 | 0.33265 | 0.41197 | 0.64655 |
| 0 | 0.68919 | 0.57902 | 0.43891 | 0.30919 |
| 0 | 0.10405 | 0.20064 | 0.27171 | 0.18765 |
| 1 | 0.41191 | 0.65387 | 0.49039 | 0.66272 |
| 0 | 0.4403 | 0.11354 | 0.14319 | 0.2209 |
| 0 | 0.51401 | 0.48766 | 0.43508 | 0.25141 |
| 0 | 0.55784 | 0.58006 | 0.81672 | 0.77214 |
| 1 | 0.67552 | 0.89131 | 0.82363 | 0.81635 |
| 1 | 0.45509 | 0.62263 | 0.34847 | 0.66168 |
| 0 | 0.71957 | 0.40107 | 0.43898 | 0.33967 |
| 1 | 0.88354 | 0.85247 | 0.77807 | 0.66199 |
| 0 | 0.58389 | 0.79749 | 0.66984 | 0.47429 |
| 1 | 0.37956 | 0.93742 | 0.82277 | 0.86062 |
| 0 | 0.56469 | 0.72725 | 0.8101 | 0.4369 |
| 1 | 0.73784 | 0.88063 | 0.69193 | 0.72766 |
| 1 | 0.27353 | 0.52464 | 0.48271 | 0.25345 |
| 1 | 0.69246 | 0.73005 | 0.61478 | 0.57218 |
| 0 | 0.51306 | 0.05197 | 0.28133 | 0.23806 |
| 1 | 0.4095 | 0.79409 | 0.82533 | 0.80774 |
| 0 | 0.14025 | 0.46707 | 0.22409 | 0.2387 |
| 1 | 0.51931 | 0.23019 | 0.27 | 0.36629 |
| 1 | 0.60018 | 0.83889 | 0.92322 | 0.83559 |
| 0 | 0.62136 | 0.54399 | 0.39336 | 0.65368 |
| 1 | 0.79698 | 0.75118 | 0.82151 | 0.53328 |
| 1 | 0.94093 | 0.88448 | 0.78143 | 0.63072 |
| 1 | 0.78841 | 0.85215 | 0.81275 | 0.93095 |
| 0 | 0.37098 | 0.1335 | 0.21598 | 0.2971 |
| 0 | 0.77665 | 0.62072 | 0.94241 | 0.84608 |
| 0 | 0.04695 | 0.08691 | 0.1522 | 0.08353 |
| 1 | 0.86195 | 0.81409 | 0.79942 | 0.62836 |
| 0 | 0.53901 | 0.36421 | 0.26555 | 0.17434 |
| 1 | 0.81589 | 0.31364 | 0.45276 | 0.67336 |
| 0 | 0.09636 | 0.44479 | 0.25893 | 0.63039 |
| 0 | 0.15705 | 0.44804 | 0.0685 | 0.25032 |
| 1 | 0.4376 | 0.35191 | 0.70233 | 0.54787 |
| 0 | 0.51779 | 0.09069 | 0.04596 | 0.11758 |
| 0 | 0.42661 | 0.19473 | 0.30974 | 0.16668 |
| 1 | 0.29265 | 0.5107 | 0.16086 | 0.36439 |
| 0 | 0.31508 | 0.39552 | 0.60125 | 0.40391 |
| 1 | 0.64539 | 0.69003 | 0.52233 | 0.40379 |
| 1 | 0.48618 | 0.42135 | 0.72654 | 0.8154 |
| 0 | 0.72003 | 0.6236 | 0.75335 | 0.51762 |
| 1 | 0.95205 | 0.92738 | 0.94407 | 0.94227 |
| 1 | 0.064 | 0.58571 | 0.45747 | 0.31111 |
| 1 | 0.28563 | 0.70936 | 0.84034 | 0.72776 |
| 1 | 0.86096 | 0.67326 | 0.88398 | 0.80021 |
| 0 | 0.28573 | 0.45257 | 0.31149 | 0.12902 |
| 1 | 0.65764 | 0.82904 | 0.82405 | 0.81884 |
| 1 | 0.68542 | 0.87685 | 0.93581 | 0.6673 |
| 1 | 0.49853 | 0.85217 | 0.74658 | 0.77888 |
| 1 | 0.38875 | 0.86225 | 0.80378 | 0.69956 |
| 1 | 0.8424 | 0.95372 | 0.78558 | 0.75263 |
| 1 | 0.70413 | 0.57549 | 0.45456 | 0.49478 |
| 0 | 0.70588 | 0.87591 | 0.83869 | 0.71854 |
| 0 | 0.81188 | 0.73632 | 0.79571 | 0.62209 |
| 1 | 0.4656 | 0.80996 | 0.74717 | 0.68755 |
| 0 | 0.17024 | 0.11443 | 0.145 | 0.13886 |
| 1 | 0.82466 | 0.83765 | 0.7649 | 0.91113 |
| 0 | 0.066 | 0.24833 | 0.16135 | 0.19252 |
| 0 | 0.77289 | 0.55741 | 0.76688 | 0.47188 |
| 1 | 0.0938 | 0.07135 | 0.10546 | 0.16012 |
| 1 | 0.60447 | 0.53913 | 0.6898 | 0.70995 |
| 0 | 0.35561 | 0.45673 | 0.29624 | 0.34375 |
| 1 | 0.33781 | 0.5794 | 0.37157 | 0.41454 |
| 1 | 0.67805 | 0.80191 | 0.65564 | 0.54083 |
| 1 | 0.59317 | 0.38238 | 0.4834 | 0.70371 |
| 0 | 0.74225 | 0.62287 | 0.65635 | 0.72543 |
| 1 | 0.14385 | 0.18308 | 0.11943 | 0.07322 |
| 1 | 0.24238 | 0.48412 | 0.31303 | 0.55003 |
| 0 | 0.12498 | 0.18761 | 0.04579 | 0.03636 |
| 1 | 0.39972 | 0.72306 | 0.80057 | 0.7811 |
| 1 | 0.14218 | 0.29767 | 0.15657 | 0.10667 |
| 1 | 0.93502 | 0.81913 | 0.68225 | 0.85159 |
| 0 | 0.51189 | 0.60115 | 0.30511 | 0.41842 |
| 1 | 0.87106 | 0.6881 | 0.90948 | 0.93955 |
| 0 | 0.08081 | 0.3545 | 0.16377 | 0.03089 |
| 0 | 0.4425 | 0.15752 | 0.10521 | 0.29703 |
| 1 | 0.63738 | 0.45305 | 0.6763 | 0.42285 |
| 0 | 0.76832 | 0.90937 | 0.85956 | 0.69285 |
| 0 | 0.08855 | 0.05607 | 0.07994 | 0.12483 |
| 1 | 0.49544 | 0.31891 | 0.57441 | 0.66599 |
| 1 | 0.89091 | 0.43562 | 0.56493 | 0.71967 |
| 0 | 0.45628 | 0.4946 | 0.42247 | 0.5511 |
| 1 | 0.69586 | 0.84969 | 0.88104 | 0.83168 |
