# Supplementary material for: Short-term risk stratification using parallel admission and reassessment features in PICU patients with infection
Source: Front Pediatr. 2026 Jun 4;14:1834603. doi: 10.3389/fped.2026.1834603 (PMC13295176; doi:10.3389/fped.2026.1834603)
Supplement: Supplementary file 3 [file Table3.docx]

Supplementary Table S3. Threshold-dependent operating characteristics of the four models in the internal test set

Random forest

| Threshold | Sensitivity | Specificity | PPV | NPV | F1 | Accuracy |
| --- | --- | --- | --- | --- | --- | --- |
| 0.10 | 0.997 | 0.083 | 0.581 | 0.962 | 0.734 | 0.595 |
| 0.20 | 0.937 | 0.236 | 0.610 | 0.747 | 0.739 | 0.629 |
| 0.30 | 0.893 | 0.369 | 0.643 | 0.730 | 0.748 | 0.662 |
| 0.40 | 0.825 | 0.465 | 0.662 | 0.676 | 0.735 | 0.667 |
| 0.50 | 0.721 | 0.598 | 0.695 | 0.627 | 0.708 | 0.667 |

LASSO

| Threshold | Sensitivity | Specificity | PPV | NPV | F1 | Accuracy |
| --- | --- | --- | --- | --- | --- | --- |
| 0.20 | 0.935 | 0.169 | 0.589 | 0.671 | 0.723 | 0.598 |
| 0.30 | 0.872 | 0.299 | 0.613 | 0.647 | 0.720 | 0.620 |
| 0.35 | 0.825 | 0.362 | 0.622 | 0.619 | 0.709 | 0.621 |
| 0.40 | 0.778 | 0.429 | 0.634 | 0.603 | 0.699 | 0.624 |
| 0.50 | 0.634 | 0.581 | 0.659 | 0.556 | 0.646 | 0.611 |

XGBoost

| Threshold | Sensitivity | Specificity | PPV | NPV | F1 | Accuracy |
| --- | --- | --- | --- | --- | --- | --- |
| 0.20 | 0.937 | 0.183 | 0.593 | 0.696 | 0.727 | 0.605 |
| 0.30 | 0.883 | 0.352 | 0.634 | 0.702 | 0.738 | 0.649 |
| 0.35 | 0.846 | 0.425 | 0.652 | 0.684 | 0.736 | 0.661 |
| 0.40 | 0.809 | 0.478 | 0.664 | 0.664 | 0.729 | 0.664 |
| 0.50 | 0.713 | 0.601 | 0.695 | 0.622 | 0.704 | 0.664 |

Stacked

| Threshold | Sensitivity | Specificity | PPV | NPV | F1 | Accuracy |
| --- | --- | --- | --- | --- | --- | --- |
| 0.20 | 0.948 | 0.223 | 0.608 | 0.770 | 0.741 | 0.629 |
| 0.30 | 0.888 | 0.392 | 0.650 | 0.733 | 0.751 | 0.670 |
| 0.35 | 0.859 | 0.468 | 0.673 | 0.723 | 0.755 | 0.687 |
| 0.40 | 0.815 | 0.508 | 0.678 | 0.683 | 0.740 | 0.680 |
| 0.50 | 0.718 | 0.615 | 0.703 | 0.631 | 0.711 | 0.673 |
