# Supplementary material for: Short-term risk stratification using parallel admission and reassessment features in PICU patients with infection
Source: Front Pediatr. 2026 Jun 4;14:1834603. doi: 10.3389/fped.2026.1834603 (PMC13295176; doi:10.3389/fped.2026.1834603)
Supplement: Supplementary file 4 [file Table4.docx]

Supplementary Table S4. Threshold-dependent operating characteristics of the random forest model in the temporal validation cohort

| Threshold | Sensitivity | Specificity | PPV | NPV | F1 | Accuracy |
| --- | --- | --- | --- | --- | --- | --- |
| 0.10 | 0.900 | 0.150 | 0.459 | 0.652 | 0.608 | 0.484 |
| 0.20 | 0.800 | 0.360 | 0.501 | 0.692 | 0.616 | 0.556 |
| 0.30 | 0.700 | 0.720 | 0.667 | 0.750 | 0.683 | 0.711 |
| 0.35 | 0.550 | 0.800 | 0.688 | 0.689 | 0.611 | 0.689 |
| 0.40 | 0.450 | 0.880 | 0.750 | 0.666 | 0.563 | 0.689 |
