# Supplementary material for: Short-term risk stratification using parallel admission and reassessment features in PICU patients with infection
Source: Front Pediatr. 2026 Jun 4;14:1834603. doi: 10.3389/fped.2026.1834603 (PMC13295176; doi:10.3389/fped.2026.1834603)
Supplement: Supplementary file 5 [file Table5.docx]

Supplementary Table S5. Candidate predictor definitions, source domains, time windows, matching patterns, and aggregation rules

| Feature name | Base feature | Window | Window definition | Source data group | Matching patterns | Aggregation rule | Unit | Clinical domain |
| --- | --- | --- | --- | --- | --- | --- | --- | --- |
| spo2_min_m0 | spo2_min | M0 | 0–6 h | Vital signs | spo2; sao2; oxygen saturation | Minimum | % | Respiratory |
| spo2_min_m1 | spo2_min | M1 | 12–36 h | Vital signs | spo2; sao2; oxygen saturation | Minimum | % | Respiratory |
| hr_max_m0 | hr_max | M0 | 0–6 h | Vital signs | heart rate; hr | Maximum | beats/min | Circulatory |
| hr_max_m1 | hr_max | M1 | 12–36 h | Vital signs | heart rate; hr | Maximum | beats/min | Circulatory |
| rr_max_m0 | rr_max | M0 | 0–6 h | Vital signs | respiratory rate; rr | Maximum | breaths/min | Respiratory |
| rr_max_m1 | rr_max | M1 | 12–36 h | Vital signs | respiratory rate; rr | Maximum | breaths/min | Respiratory |
| fio2_max_m0 | fio2_max | M0 | 0–6 h | Vital signs | fio2; fraction of inspired oxygen | Maximum | % | Respiratory support |
| fio2_max_m1 | fio2_max | M1 | 12–36 h | Vital signs | fio2; fraction of inspired oxygen | Maximum | % | Respiratory support |
| wbc_m0 | wbc | M0 | 0–6 h | Laboratory tests | wbc; white blood cell | Last | 10^9/L | Inflammation |
| wbc_m1 | wbc | M1 | 12–36 h | Laboratory tests | wbc; white blood cell | Last | 10^9/L | Inflammation |
| neut_pct_m0 | neut_pct | M0 | 0–6 h | Laboratory tests | neutrophil; neut% | Last | % | Inflammation |
| neut_pct_m1 | neut_pct | M1 | 12–36 h | Laboratory tests | neutrophil; neut% | Last | % | Inflammation |
| lymph_pct_m0 | lymph_pct | M0 | 0–6 h | Laboratory tests | lymphocyte; lymph% | Last | % | Inflammation |
| lymph_pct_m1 | lymph_pct | M1 | 12–36 h | Laboratory tests | lymphocyte; lymph% | Last | % | Inflammation |
| crp_m0 | crp | M0 | 0–6 h | Laboratory tests | crp; c-reactive protein | Last | mg/L | Inflammation |
| crp_m1 | crp | M1 | 12–36 h | Laboratory tests | crp; c-reactive protein | Last | mg/L | Inflammation |
| pct_m0 | pct | M0 | 0–6 h | Laboratory tests | procalcitonin; pct | Last | ng/mL | Inflammation |
| pct_m1 | pct | M1 | 12–36 h | Laboratory tests | procalcitonin; pct | Last | ng/mL | Inflammation |
| alb_m0 | alb | M0 | 0–6 h | Laboratory tests | albumin; alb | Last | g/L | Biochemistry |
| alb_m1 | alb | M1 | 12–36 h | Laboratory tests | albumin; alb | Last | g/L | Biochemistry |
| alt_m0 | alt | M0 | 0–6 h | Laboratory tests | alt; alanine aminotransferase | Last | U/L | Biochemistry |
| alt_m1 | alt | M1 | 12–36 h | Laboratory tests | alt; alanine aminotransferase | Last | U/L | Biochemistry |
| ast_m0 | ast | M0 | 0–6 h | Laboratory tests | ast; aspartate aminotransferase | Last | U/L | Biochemistry |
| ast_m1 | ast | M1 | 12–36 h | Laboratory tests | ast; aspartate aminotransferase | Last | U/L | Biochemistry |
| tbil_m0 | tbil | M0 | 0–6 h | Laboratory tests | total bilirubin; tbil | Last | μmol/L | Biochemistry |
| tbil_m1 | tbil | M1 | 12–36 h | Laboratory tests | total bilirubin; tbil | Last | μmol/L | Biochemistry |
| crea_m0 | crea | M0 | 0–6 h | Laboratory tests | creatinine; crea | Maximum | μmol/L | Renal |
| crea_m1 | crea | M1 | 12–36 h | Laboratory tests | creatinine; crea | Maximum | μmol/L | Renal |
| bun_m0 | bun | M0 | 0–6 h | Laboratory tests | bun; blood urea nitrogen | Maximum | mmol/L | Renal |
| bun_m1 | bun | M1 | 12–36 h | Laboratory tests | bun; blood urea nitrogen | Maximum | mmol/L | Renal |
| na_sod_m0 | na_sod | M0 | 0–6 h | Laboratory tests | sodium; na | Last | mmol/L | Electrolyte |
| na_sod_m1 | na_sod | M1 | 12–36 h | Laboratory tests | sodium; na | Last | mmol/L | Electrolyte |
| k_m0 | k | M0 | 0–6 h | Laboratory tests | potassium; k | Last | mmol/L | Electrolyte |
| k_m1 | k | M1 | 12–36 h | Laboratory tests | potassium; k | Last | mmol/L | Electrolyte |
| cl_m0 | cl | M0 | 0–6 h | Laboratory tests | chloride; cl | Last | mmol/L | Electrolyte |
| cl_m1 | cl | M1 | 12–36 h | Laboratory tests | chloride; cl | Last | mmol/L | Electrolyte |
| ca_m0 | ca | M0 | 0–6 h | Laboratory tests | calcium; ca | Last | mmol/L | Electrolyte |
| ca_m1 | ca | M1 | 12–36 h | Laboratory tests | calcium; ca | Last | mmol/L | Electrolyte |
| ph_m0 | ph | M0 | 0–6 h | Blood gas | ph | Minimum | unitless | Blood gas |
| ph_m1 | ph | M1 | 12–36 h | Blood gas | ph | Minimum | unitless | Blood gas |
| pco2_m0 | pco2 | M0 | 0–6 h | Blood gas | pco2 | Maximum | mmHg | Blood gas |
| pco2_m1 | pco2 | M1 | 12–36 h | Blood gas | pco2 | Maximum | mmHg | Blood gas |
| po2_m0 | po2 | M0 | 0–6 h | Blood gas | po2; pao2 | Minimum | mmHg | Blood gas |
| po2_m1 | po2 | M1 | 12–36 h | Blood gas | po2; pao2 | Minimum | mmHg | Blood gas |
| hco3_m0 | hco3 | M0 | 0–6 h | Blood gas | hco3; bicarbonate | Last | mmol/L | Blood gas |
| hco3_m1 | hco3 | M1 | 12–36 h | Blood gas | hco3; bicarbonate | Last | mmol/L | Blood gas |
| lactate_m0 | lactate | M0 | 0–6 h | Blood gas | lactate | Maximum | mmol/L | Metabolism |
| lactate_m1 | lactate | M1 | 12–36 h | Blood gas | lactate | Maximum | mmol/L | Metabolism |

Table note. M0 denotes the admission window from 0 to 6 hours after PICU admission, and M1 denotes the early reassessment window from 12 to 36 hours after admission. Matching patterns were applied in a case-insensitive manner to harmonize semantically related raw variable labels across source tables. Aggregation rules were prespecified according to the anticipated direction of physiologic deterioration and the intended interpretation of each variable within a given window. Minimum values were used for indicators in which lower values reflect greater physiologic compromise, maximum values for indicators in which higher values reflect greater severity, and the last available value for variables intended to represent end-of-window status.

Abbreviations. SpO2, peripheral oxygen saturation; HR, heart rate; RR, respiratory rate; FiO2, fraction of inspired oxygen; WBC, white blood cell count; CRP, C-reactive protein; PCT, procalcitonin; ALB, albumin; ALT, alanine aminotransferase; AST, aspartate aminotransferase; TBIL, total bilirubin; CREA, creatinine; BUN, blood urea nitrogen; Na, sodium; K, potassium; Cl, chloride; Ca, calcium; HCO3, bicarbonate.
