# Supplementary material for: Short-term risk stratification using parallel admission and reassessment features in PICU patients with infection
Source: Front Pediatr. 2026 Jun 4;14:1834603. doi: 10.3389/fped.2026.1834603 (PMC13295176; doi:10.3389/fped.2026.1834603)
Supplement: Supplementary file 6 [file Table6.docx]

Supplementary Table S6 Hyperparameter search space and final selected parameters for the candidate models

| Model | Hyperparameter | Search space | Final selected value |
| --- | --- | --- | --- |
| LASSO logistic regression | Penalty | Fixed as L1 | L1 |
| LASSO logistic regression | Solver | Fixed as saga | saga |
| LASSO logistic regression | C | 0.01, 0.03, 0.1, 0.3, 1, 3, 10 | 0.3 |
| LASSO logistic regression | Class weight | Fixed as balanced | balanced |
| LASSO logistic regression | Maximum iterations | Fixed as 5000 | 5000 |
| Random forest | Number of trees (n_estimators) | 300, 500, 800 | 500 |
| Random forest | Maximum depth (max_depth) | None, 4, 6, 8, 10 | None |
| Random forest | Minimum samples to split (min_samples_split) | 2, 5, 10 | 2 |
| Random forest | Minimum samples per leaf (min_samples_leaf) | 1, 2, 4 | 1 |
| Random forest | Maximum features (max_features) | sqrt, 0.3, 0.5, None | sqrt |
| Random forest | Class weight | Fixed as balanced | balanced |
| XGBoost | Number of trees (n_estimators) | 150, 250, 400 | 250 |
| XGBoost | Maximum depth (max_depth) | 2, 3, 4, 5 | 3 |
| XGBoost | Learning rate (learning_rate) | 0.03, 0.05, 0.1 | 0.05 |
| XGBoost | Row subsampling (subsample) | 0.7, 0.8, 1.0 | 0.8 |
| XGBoost | Column subsampling (colsample_bytree) | 0.6, 0.8, 1.0 | 0.8 |
| Stacked model | Meta-learner | Fixed as logistic regression | logistic regression |
| Stacked model | Base learners | Fixed as LASSO, random forest, and XGBoost | LASSO, random forest, and XGBoost |

Hyperparameter tuning was performed within the training data only. For LASSO logistic regression, the inverse regularization strength parameter C was tuned across a prespecified grid while penalty, solver, class weighting, and iteration limit were fixed in advance. For the random forest and XGBoost models, grid-based search was conducted over the candidate parameter combinations listed above. The stacked model used logistic regression as the meta-learner and combined the predictions from the LASSO, random forest, and XGBoost base models. Final selected values refer to the parameter set retained for model development after internal tuning.
