# Supplementary material for: Short-term risk stratification using parallel admission and reassessment features in PICU patients with infection
Source: Front Pediatr. 2026 Jun 4;14:1834603. doi: 10.3389/fped.2026.1834603 (PMC13295176; doi:10.3389/fped.2026.1834603)
Supplement: Supplementary file 7 [file Table7.docx]

Supplementary Table S7. Prespecified exclusion and preprocessing rules

| Stage | Variable or rule | Status | Illustrative count | Reason |
| --- | --- | --- | --- | --- |
| Cohort assembly | Age outside 0–18 years | Removed | 0 admissions removed | Did not meet the prespecified age eligibility criterion |
| Cohort assembly | Duplicate PICU stays for the same patient | One retained | 34 admissions removed | The first eligible PICU admission was retained |
| Data cleaning | Admissions with >70% missing core predictors | Removed | 24 admissions removed | Prespecified admission-level missingness threshold |
| Data cleaning | Variables with >50% missingness | Removed | 8 variables removed | Prespecified variable-level missingness threshold |
| Data cleaning | Constant predictors | Removed | 2 variables removed | Non-informative predictor |
| Data cleaning | Near-zero variance predictors | Removed | 3 variables removed | Minimal classification value |
| Data cleaning | One variable from each highly collinear pair (\|r\| ≥ 0.90) | Removed | 4 variables removed | The more clinically interpretable or less-missing variable was retained |
| Reporting / model specification | Age band | Kept | 1 variable retained | Prespecified fixed covariate retained in all models |
